# Accelerated Training of Skilled Birth Attendants in a Marginalized Population on the Thai-Myanmar Border: A Multiple Methods Program Evaluation

**DOI:** 10.1371/journal.pone.0164363

**Published:** 2016-10-06

**Authors:** Adrienne Lynne White, Thaw Htwe Min, Mechthild M. Gross, Ladda Kajeechiwa, May Myo Thwin, Borimas Hanboonkunupakarn, Hla Hla Than, Thet Wai Zin, Marcus J. Rijken, Gabie Hoogenboom, Rose McGready

**Affiliations:** 1 Pregnancy Research Centre, The Royal Women’s Hospital, Melbourne, Australia; 2 Shoklo Malaria Research Unit, Mahidol-Oxford Tropical Medicine Research Unit, Faculty of Tropical Medicine, Mahidol University, Mae Sot, Thailand; 3 Midwifery Research and Education Unit, Department of Obstetrics, Gynaecology & Reproductive Medicine, Hannover Medical School, Hannover, Germany; 4 Mahidol-Oxford Tropical Medicine Research Unit, Department of Clinical Tropical Medicine, Faculty of Tropical Medicine, Mahidol University, Bangkok, Thailand; 5 Julius Global Health and Department of Woman and Baby, University Medical Centre Utrecht, Utrecht, The Netherlands; 6 Centre for Tropical Medicine and Global Health, Nuffield Department of Clinical Medicine, University of Oxford, Oxford, United Kingdom; Royal Children's Hospital, AUSTRALIA

## Abstract

**Background:**

To evaluate a skilled birth attendant (SBA) training program in a neglected population on the Thai-Myanmar border, we used multiple methods to show that refugee and migrant health workers can be given effective training in their own environment to become SBAs and teachers of SBAs. The loss of SBAs through resettlement to third countries necessitated urgent training of available workers to meet local needs.

**Methods and Findings:**

All results were obtained from student records of theory grades and clinical log books. Qualitative evaluation of both the SBA and teacher programs was obtained using semi-structured interviews with supervisors and teachers. We also reviewed perinatal indicators over an eight-year period, starting prior to the first training program until after the graduation of the fourth cohort of SBAs.

**Results:**

Four SBA training programs scheduled between 2009 and 2015 resulted in 79/88 (90%) of students successfully completing a training program of 250 theory hours and 625 supervised clinical hours. All 79 students were able to: achieve pass grades on theory examination (median 80%, range [70–89]); obtain the required clinical experience within twelve months; achieve clinical competence to provide safe care during childbirth. In 2010–2011, five experienced SBAs completed a train-the-trainer (TOT) program and went on to facilitate further training programs. Perinatal indicators within Shoklo Malaria Research Unit (SMRU), such as place of birth, maternal and newborn outcomes, showed no significant differences before and after introduction of training or following graduate deployment in the local maternity units. Confidence, competence and teamwork emerged from qualitative evaluation by senior SBAs working with and supervising students in the clinics.

**Conclusions:**

We demonstrate that in resource-limited settings or in marginalized populations, it is possible to accelerate training of skilled birth attendants to provide safe maternity care. Education needs to be tailored to local needs to ensure evidence-based care of women and their families.

## Introduction

The Office of the United Nations High Commissioner for Refugees (UNHCR) estimates that in March 2016 there were 45.2 million forcibly displaced people around the world of whom 15.4 million are refugees [1]. It is also estimated that in 2010 more than 60 million women gave birth without care leaving them vulnerable to birth complications [2–4].

Most maternal deaths and disability are preventable if women have access to care from a skilled birth attendant (SBA) [5–7]. The State of the World’s Midwives report recommends improving the status, education and regulation of midwives to improve care of mothers and babies [6, 8]. There is a shortage of qualified midwives world-wide [9] and it is recognised that midwives and other SBAs play a central role in the reduction of maternal and newborn mortality and morbidity, particularly in neglected populations and resource-limited settings [7, 8, 10, 11].

The World Health Organization (WHO) defines a skilled birth attendant as: *an accredited health professional*, *such as a midwife*, *doctor or nurse*, *who has been educated and trained to proficiency in the skills needed to manage normal (uncomplicated) pregnancies*, *childbirth and the immediate postnatal period*, *and in the identification*, *management and referral of complications in women and newborns* [5]. The WHO lists core skills that SBAs must have and states that further skills may be added to meet the requirements of the setting [5]. The skills needed to provide basic emergency obstetric care for mothers and newborns (BEmONC) has also been recommended as the focus of training for SBAs [12]. In resource-rich countries, competence to practice as a midwife is assessed during and following an accredited midwifery education program and by registration authorities to assess continued fitness to practice [13, 14]. In populations without formal infrastructure, such as in refugee camps, training and assessment of baccalaureate-level midwives is neither feasible nor possible and there are a limited number of publications on the training of birth attendants in such settings [4, 15–20]. In refugee settings, areas of conflict, or where national systems have collapsed, SBA training must be adapted to local conditions and accelerated to provide immediate care to vulnerable populations [21, 22]. In this setting it is also important to provide resources, competent teachers and an enabling environment, both during and following basic training [9, 10, 23, 24]. Many refugee health facilities also lack obstetricians and must rely on non-government organization (NGO) staff or hired consultants to meet short-term medical staffing needs [4]. This study describes the evaluation of an accelerated skilled birth attendant (SBA) curriculum designed to meet the specific requirements of refugee and migrant populations from Myanmar (Burma) giving birth in the Shoklo Malaria Research Unit (SMRU) clinics located along the north-western border between Thailand and Myanmar.

## Methods

### Setting

On the western border of Thailand in Tak Province there is an estimated population of 140,000 people from Myanmar living in refugee camps and another estimated 200,000 migrant workers from Myanmar living in Thailand, most of who identify as either ethnic Karen or Burman and speak Burmese, several Karen dialects and sometimes English and Thai. Neglect of the education system in Myanmar and lack of recognition of the NGO-run refugee camp schools’ certification combined with limited higher education opportunities [25, 26] has affected achievement levels of school leavers in the area for decades [26, 27]. Most students aim to complete the 10^th^ Standard exam at around 16 years of age in Myanmar or in the camps.

SMRU, located on the Thai side of the Thai-Myanmar border, is an operational field-based research unit that has provided free, basic humanitarian care to refugees since 1986 and in migrant communities since 1998, within their five clinics (S1 Fig. **Map of SMRU Clinics)**. In Maela refugee camp, Première Urgence-Aide Médicale International (PU-AMI) is the major health provider. In contrast to NGO support for refugee health, migrant health care is not subsidized, with policy makers in Thailand grappling with how to pay for migrant health [28, 29].

In 1986, in this population, 75% of births took place at home with traditional birth attendants (TBAs) with no formal training. TBAs still provide support to women but through a process of community encouragement more than 75% of women now choose to give birth at SMRU [30]. Pregnancy care has been a major focus of SMRU health care provision due to high levels of maternal [30, 31] and infant mortality [32, 33]. SMRU provides care for uncomplicated and assisted vaginal births and post-partum care of mothers and babies including those needing special care [32]. Each year about 2,500 women attend SMRU antenatal clinics and most give birth at SMRU clinics in either Mae La refugee camp, Wang Pha or Mawker Thai migrant clinics (S1 Fig. **Map of SMRU Clinics)**. Care is provided by locally trained staff using evidence-based protocols with 24-hour medical back up via telephone. The WHO partogram has been used since 1995 [34, 35] and women with complex problems or who need caesarean delivery are transferred to Thai hospitals between 30 and 75 minutes away by car. SMRU funds care in Thailand hospitals. SMRU maternity clinics are able to provide all seven signal functions for Basic Emergency Obstetric and Newborn Care (BEmONC), parental administration of an oxytocic, antibiotics and anticonvulsants, removal of retained products of conception, assisted vaginal birth including breech birth, resuscitation of the newborn using a bag and mask and screened blood transfusions [23, 36]. All birth records at SMRU are computer based and stored at SMRU head office in Mae Sot. A chronic shortage of SBAs prompted SMRU to develop a formal training curriculum that started in 2009.

### Curriculum development

After consultation with local SBAs, SMRU invited a registered midwife to develop a SBA curriculum. SBA core competencies from the International Confederation of Midwives (ICM), International Federation of Obstetricians and Gynaecologists (FIGO) and WHO [5] were used as a basis to develop a 250 hour theory curriculum adapted as needed for local conditions [37]. The WHO guide to Integrated Management of Pregnancy and Childbirth (IMPAC) 2006 [38] was also used to inform the curriculum content and both documents were used to update the local clinical practice guidelines (SMRU Obstetric Manual, 2007, 2^nd^ Edition, SMRU Paediatric Manual 2008, 3rd edition) with reviews by local SBAs, expatriate and local doctors for culturally appropriate content.

The curriculum was designed as a fit-for-purpose apprenticeship model with classes scheduled to avoid unnecessary disruption to clinical care. Lectures in simple English were given on PowerPoint^™^ with printed notes given to students at no cost to them. Students had no access to the Internet, and limited access to other resources such as textbooks. Close proximity of the classrooms to the maternity units at each site enabled both scheduled and opportunistic bedside teaching and demonstrations.

The clinical component of a minimum of 625 hours of supervised (by local SBAs and expatriate medical practitioners) clinical practice was competency based, equally weighted to the theory curriculum for assessment purposes, and scheduled over six to twelve months. A comprehensive logbook in English was designed as a record of clinical placements, clinical experiences, reflection on practice and achievement of competence (S1 File. **SBA clinical practice logbook**).

Preference was given to applicants who were at least eighteen years old with no upper age limit. Each applicant was required to have completed (but may not have passed) high school education to year 10; write an essay in English on motivation to study as a SBA and attend a formal interview. For the 2013 and 2014 intakes, potential students also completed a three-week, introduction to health-care course with subjects such as basic science, anatomy, physiology, patient assessment skills and principles of infection control. The SBA course was offered free, but on recruitment students were asked to sign a non-binding agreement to work at SMRU for a year after their graduation. In 2012, attempts were made to further strengthen recruitment strategies and prevent post-graduation attrition by requiring students to have verbal family support for their study, and by providing incentives to remain at SMRU such as free accommodation.

To encourage sustainability of the training program students were employed full-time (six days per week); SBAs involved with mentorship and student supervision were advantaged in any application for promotion; and a SBA train-the-trainer (TOT) program was introduced during the second year. Skill recognition and up-skilling of existing SBAs was also achieved concurrently with these two programs.

### Evaluation components

To comprehensively evaluate the SBA curriculum a multiple methods approach with both quantitative and qualitative components was chosen [39].

#### Quantitative

The quantitative components evaluated included: completion and employment of students from the first four cohorts (2009, 2010, 2013 and 2014); theory exam results; completed clinical skills and perinatal indicators before and since the SBA program commenced.

The numbers of students graduating and attrition following each program were evaluated to ascertain the need for changes to recruitment, employment conditions and curriculum restructure. The knowledge-based (theory) component of the curriculum was assessed in English by three written Multiple Choice Question (MCQ) exams of 100 questions each with a pass grade set at 60% and final theory score calculated as the average of the three MCQ exams. MCQ theoretical assessment was chosen to minimise disadvantage to students with limited English and/or Burmese language skills [17]. Each exam was translated into written Burmese by a medical doctor and verified by independent back-translation. Differences were amended by bringing both the forward-translator and the back-translator to a single meeting. If required during the exam, Karen-speaking SBA teachers offered spoken translation of questions for students not fluent in either English or Burmese. Standardised clinical assessment tools were designed for this population and to reduce subjectivity in assessment, with simulations if clinical cases were not available [39].

Clinical assessment of knowledge, skills and attitudes to reach competence included comparison with the ICM competencies for international midwifery practice [9] and requirements by regulation and registration authorities in Australia [40] and UK [41]. Assessment comprised logbook records of clinical hours; a minimum number of supervised clinical experiences (such as antenatal visits, uncomplicated births and postnatal care); assessment of ten core competencies; a summary of the student’s continuity of care case study and a diary of reflection on practice. (S1 File. **SBA clinical practice logbook**). Students were clinically assessed by SMRU trained and experienced SBAs, SBA teachers, local and expatriate doctors, and for the first two groups, by an expatriate registered midwife (AW).

To obtain an assessment of birth room safety pre-and post-SBA training, perinatal indicators were compared for an eight-year time period from 2008–2015. This corresponds to the year before the SBA student workforce commenced practical duties and each year thereafter until 2015. The perinatal indicators included place of birth, maternal deaths, stillbirths and neonatal deaths, Apgar Scores and the need for neonatal resuscitation. Rates of induction of labour, augmentation of labour, post-partum haemorrhage, episiotomies and use of a partogram were also included.

#### Qualitative

Two program components were evaluated qualitatively; the SBA curriculum and the TOT program. One to one semi-structured interviews rather than focus groups were chosen to provide privacy and confidentiality and due to the complex logistics required to bring participants together in one place. Participation was voluntary, written and verbal consent was given prior to interviews and SBAs were free to withdraw at any time without disadvantage. Although no longer working at SMRU at the time of the interviews, the researcher was well known to all participants, which had the advantage of trust but the disadvantage of positive bias. Interviews were scheduled at times to suit the interviewees during January 2014. Interview length was determined by the interviewee and lasted between 5 and 25 minutes. The researcher made hand written notes of the conversation and transcribed them into a Word document on the day of each interview. Participants were given the opportunity to verify interview transcripts typed in English for correct capture of responses [42]. All responses were de-identified and names changed to ensure confidentiality.

For evaluation of the SBA curriculum, five experienced local SBAs from the three birth units agreed to participate in semi-structured interviews in English in January 2014. All five SBAs invited to participate had supervised SBA students since before the start of the first SBA training, during each training and for at least one year after the completion of the fourth SBA program. They were asked the following open-ended questions: 1, ‘Tell me about SBA training and how it has changed how things work in delivery [birth room]’. 2, ‘How is it [SBA practice] different now at SMRU compared to how it was before SBA training?’

Evaluation of the TOT program was conducted two years after it was completed. Of the five teachers, one had left the organization and three of the four remaining teachers agreed to participate in in semi-structured interviews, in English, with one on leave in Myanmar unable to participate. Open-ended questions were used to elicit information, for example: 1, ‘You have been an experienced teacher for many years, but now you have completed TOT how do you feel about teaching?’ Or, 2, ‘How do you feel about teaching now compared to before TOT?’

### Ethics statement

For the extraction of data, ethical approval for retrospective analysis of pregnancy records was given by the Oxford Tropical Research Ethics Committee (OXTREC 28–09, amended 19 April 2012). For the semi-structured interviews with SBAs providing feedback on the program, written informed consent was obtained at the time of time of the interviews (no minors were interviewed). Permission to study these transcripts and evaluate the program in general was obtained locally from the Tak Community Advisory Board TCAB-13/2/2015.

### Data analysis

Quantitative data were analysed using SPSS for Windows^™^ (Version 20, SPSS Inc.). Continuous normally distributed data were described by their mean and non-normally distributed data by their median and inter-quartile range. Percentages were calculated for categorical data, which were compared using the χ² test or Fisher’s exact test. Student’s *t* test or the Mann-Whitney U tests were used to compare continuous variables. A high score for the theory exam was set at ≥85.0%.

Qualitative data of transcribed interviews were analysed by two researchers, with transcriptions sorted into meaning units and major themes were identified. Any rating disagreements between the researchers were resolved by discussion.

## Results

### Curriculum outcomes

Between October 2009 and August 2015, 88 women started SBA training in four separate cohorts (Table 1). A total of 79/88 (90%) have graduated as SBAs with the fourth cohort graduating in August 2015. Of the 9 students who dropped out of training prior to completion of all requirements only two were failing the theory or clinical components of the program and chose to leave rather than attempting to gain pass standard. Reasons given for dropping out are listed in Table 1.

**Table 1:** Training and workforce retention for each SBA training group.

	Group 1 n(%)n = 22	Group 2 n(%)n = 24	Group 3 n(%)n = 21	Group 4 n(%)n = 21	Total n(%)n = 88
Start date (dd-mmm-yy)	16-Oct-09	28-Oct-10	01-Apr-13	11-Aug-14	
Dropped out of course	3 (14)	1 (4)	3 (14)	2 (9)	9 (10)
Reasons					
*Marriage/pregnancy*	1	1	1^	1	4
*Family problem*	1^	-	1	-	2
*Further education in Myanmar*	-	-	1	-	1
*Serious Illness*	1	-	-	-	1
*Resettled to a third country*	-	-	-	1	1
Completed training	19 (86)	23 (96)	18 (86)	19 (91)	79 (90)
Working at SMRU ≥12mths	15 (68)	21 (88)	15 (71)	14 (66)	65 (74)
Completed training and still working SMRU (as at Mar-16)	8 (36)	7 (29)	14 (67)	13 (62)	42 (48)

^ failing on practical or theory when dropped out.

#### Student retention and promotion

Most of the SBA graduates 65/88 (74%) continued to work at SMRU for at least 12 months. However, 5.5 years later, fewer than half the graduates 42/88 (48%) were still working at SMRU. Reasons for this attrition are given in Table 2. The main reasons for attrition are given as returning to Myanmar and obtaining work elsewhere.

**Table 2:** Reasons for SBA graduate attrition from the workforce (2009–2016).

	Group 1 n(%)n = 11	Group 2 n(%)n = 16	Group 3 n(%)n = 4	Group 4 n(%)n = 6	Total n(%)n = 37
Return to Myanmar	4 (36)	4 (25)	4 (25)	0	9 (24)
Employment elsewhere	1 (9)	3 (19)	3 (75)	1 (14)	8 (22)
Health or family issues	2 (18)	3 (19)	0	1 (17)	6 (16)
Further study elsewhere	3 (27)	2 (12)	0	0	5 (13)
No local positions available	0	0	0	4 (57)	4 (11)
Migration to a third country	0	3 (19)	0	0	3 (8)
Dissatisfaction with their position	1 (9)	1 (6)	0	0	2 (5)

The proportion of SBAs who completed the requirements for promotion or who were progressing towards promotion were also reviewed (Table 3). Nearly half 28/60 (47%) of those who graduated from the first three groups were in the process of achieving promotion with two reaching senior positions.

**Table 3:** The proportion of graduates attaining more senior SBA positions in total and at SMRU.

	Group 1 n = 19		Group 2 n = 23		Group 3 n = 18		Total n = 60	
Position	Total (n)	SMRU (n)	Total (n)	SMRU (n)	Total (n)	SMRU (n)	Total (n)	SMRU (n)
Training for junior^**#**^	0	0	4	3	12	12	18	15
Reached junior	6	4	5	2	2	2	11	8
Training for senior*	2	1	5	2	0	0	7	3
Reached senior	2	2	0	0	0	0	2	2
Total advancing	10	7	14	7	14	14	38	28
Advanced/-ing as at March 2016 n (%)	10(53)	7(37)	14(61)	7(30)	14(78)	14(78)	38(63)	28(47)

Total = total of all graduates; SMRU = total of all graduates still employed at SMRU

^**#**^Junior = on completion of a graduate year and further competencies awarded a higher salary.

*Senior = on completion of advanced practice competencies awarded a higher salary and is able to supervise others.

#### Theory results

The trends in demographics of the students and the median scores achieved on the theory component have been summarized in Table 4. Most students were able to achieve pass marks on MCQ without the need to re-sit. One failing student was found to have language difficulties and following verbal translation of the exam questions into her dialect she achieved over 90%.

**Table 4:** Demographic characteristics of SBA students who completed training at SMRU 2009–15.

Characteristics	Group 1 n = 19	Group 2 n = 23	Group 3 n = 18	Group 4 n = 19
Age in yrs., mean±SD [min-max]	27±9 [19–42]	23±4 [17–32]	23±3 [18–28]	24±2 [19–29]
Aged ≥ 30 yrs., n (%)	6 (32)	2 (9)	0	0
Number of languages spoken, n (%)				
Two	5 (26)	4 (17)	2 (11)	8 (40)
Three	11 (58)	18 (78)	12 (67)	12 (60)
Four	3 (16)	1 (4)	4 (22)	0
English ability Good, (n) %	4 (21)	6 (26)	7 (39)	6 (32)
Theory median score [IQR*]	81 [76–86]	77 [75–89]	81 [75–85]	79 [70–83]

Abbreviations: SD standard deviation, IQR inter-quartile range.

The median number of languages and age of participants did not differ significantly for students who achieved a high (≥85%) compared to a lower theory score (data not shown). For groups 3 and 4 each student’s 10^th^ Standard education results were available. Students who had not passed 10^th^ standard education were still able to pass the SBA training. There was no statistically significant difference in the proportion who achieved high scores (≥85% on the theory) according to whether they had 7/21 (33%) or had not passed 2/16 (13%), their 10^th^ Standard examination, and p = 0.248, although this conclusion needs to be interpreted with caution due to the low numbers.

#### Clinical competency results

The median number of supervised episodes of care of mothers and babies achieved by students during the twelve months was high (Table 5). Graduating SBAs have achieved ICM essential competencies for basic midwifery practice and in many instances have exceeded the number of episodes of care specified for midwifery registration in Australia and UK [40, 41]. All students were able to complete the clinical requirements of the program to achieve competence within the 12 months allocated (Table 5).

**Table 5:** Median (minimum* and maximum) number of clinical skills achieved per group in graduating students.

	Group 1n = 19 Median (min-max)	Group 2n = 23	Group 3n = 18	Group 4n = 19	United Kingdom requirements for registration [41]	Australian requirements for registration [40]
First antenatal visit	10 (3–17)	30 (20–42)	20 (11–30)	31 (16–72)	Antenatal visits = 100	Antenatal visits = 100
Return antenatal visit	76 (43–119)	80 (60–197)	60 (59–120)	81 (60–160)		
Complex antenatal care	68 (31–158)	75 (50–126)	50 (32–107)	56 (37–83)	40	40
Ultrasound observed	18 (3–42)	27 (20–44)	22 (10–34)	22 (6–34)	Not specified	Not specified
Vaginal examination	30 (8–69)	102 (79–140)	75 (46–141)	93 (38–168)	Not specified	Not specified
Repair of perineum	5 (0–22)	25 (6–41)	12 (1–40)	12 (7–13)	Not specified	Not specified
Uncomplicated birth	32 (21–64)	45 (33–59)	43 (13–55)	40 (24–52)	40, or 30 normal + 20 assist	30 normal + 10 assist
Complex birth (assist)	55 (30–148)	55 (40–114)	46 (7–107)	43 (18–75)	40 including complex ANC/PNC	40
Reception of newborn	20 (9–41)	41 (33–70)	34 (10–53)	38 (24–59)	Included in birth care	20
Postnatal care	26 (9–51)	48 (34–63)	35 (19–62)	40 (19–61)	100	100
Complex postnatal care	26 (11–59)	53 (44–71)	44 (9–80)	44 (14–86)		
Family planning advice	12 (5–36)	34 (26–48)	22 (10–55)	33 (9–53)	Included in postnatal care	Included in postnatal care
Sick newborns (observed)	10 (4–17)	22 (17–99)	15 (6–60)	19 (3–41)	Not specified	Not specified

Min: Minimum, Max: Maximum,

*Minimum/maximum number of competencies for group one may underestimate experience, as some students did not record all episodes of care.

#### Perinatal indicators of birth room safety

Major indicators from 2008, the year prior to commencement of SBA training, through 2009–2015, show an increased proportion of antenatal care attenders birthing with SBAs at SMRU, and no deterioration in major indicators (Table 6). A univariate comparison of indicator data from the year 2008 and the year 2015, suggests significant improvement in most indicators including an increase in the proportion of women birthing at SMRU, having partograms completed and a decrease in the proportion of stillbirth.

**Table 6:** Perinatal indicators before (2008) and after introduction of SBA training programs (2009–2015).

	2008	2009	2010	2011	2012	2013	2014	2015	P-value^e^
Birthing women^a^	n = 2511 n(%)	n = 2735 n(%)	n = 2668 n(%)	n = 2713 n(%)	n = 2708 n(%)	n = 2585 n(%)	n = 2590 n(%)	n = 2663 n(%)	
SMRU	1436 (57.2)	1632 (59.7)	1784 (66.9)	2083 (76.8)	2210 (81.6)	2100 (81.2)	2155 (83.2)	2181 (81.9)	<0.001
Home	675 (26.9)	710 (26.0)	582 (21.8)	336 (12.4)	281 (10.4)	242 (9.4)	225 (8.7)	235 (8.8)	<0.001
Hospital	312 (12.4)	292 (10.7)	223 (8.4)	233 (8.6)	199 (7.0)	217 (8.4)	193 (7.5)	229 (8.6)	<0.001
Other	88 (3.5)	101 (3.7)	79 (3.0)	61 (2.2)	27 (1.0)	25 (1.0)	17 (0.7)	18 (0.7)	<0.001
Singletons	2489 (99.1)	2707 (99.0)	2637 (98.8)	2687 (99.0)	2687 (99.2)	2561 (99.1)	2536 (97.9)	2609 (98.0)	0.001
C-section	100 (4.0)	110 (4.1)	112 (4.2)	119 (4.4)	121 (4.5)	143 (5.6)	125 (4.9)	161 (6.2)	0.003
Maternal death^b^	0	0	0	0	0	0	1^c^	0	n.a
**SMRU singletons**	1426	1617	1767	2065	2194	2088	2125	2145	
Partogram done	1263 (87.6)	1593 (98.5)	1661 (94.0)	1986 (96.2)	2163 (98.6)	2067 (99.0)	2120 (99.7)	2140 (99.8)	<0.001
Stillbirths	14 (1.0)	11 (0.7)	18 (1.0)	17 (0.8)	19 (0.9)	14 (0.7)	14 (0.7)	9 (0.4)	0.0276
Induction	35 (2.7)	81 (5.0)	129 (7.3)	159 (7.7)	108 (4.9)	124 (5.9)	125 (5.9)	108 (5.0)	<0.001
Augmentation	71 (5.5)	160 (9.9)	251 (14.2)	317 (15.4)	291 (13.2)	247 (11.8)	232 (11)	187 (8.7)	<0.001
PPH	68 (5.3)	114 (7.0)	140 (7.9)	162 (7.9)	87 (4.0)	101 (4.8)	137 (6.4)	175 (8.2)	<0.001
Apgar 1 min^d^	8 [2–9]	9 [1–9]	9 [1–10]	9 [1–10]	9 [1–10]	9 [1–10]	9 [1–10]	9 [1–10]	n.a
Apgar 5 min^d^	9 [5–10]	10 [1–10]	10 [2–10]	10 [1–10]	10 [1–10]	10 [1–10]	10 [1–10]	10 [2–10]	n.a
Neonatal resuscitation^d^	56 (4.4)	59 (3.7)	59 (3.4)	73 (3.6)	48 (2.2)	43 (2.1)	68 (3.3)	69 (3.3)	0.090
Early neonatal death^d^	14 (1.1)	18 (1.1)	19 (1.1)	16 (0.8)	11 (0.5)	6 (0.3)	8 (0.4)	8 (0.4)	0.013
Episiotomy	152 (12.2)	68 (4.3)	76 (4.6)	122 (6.2)	112 (5.2)	117 (5.7)	114 (5.4)	115 (5.4)	<0.0001
Episiotomy if parity 0	140 (29.1)	63 (10.9)	68 (11.0)	106 (15.4)	104 (13.0)	105 (13.3)	99 (12.3)	105 (12.7)	<0.001

^a^ EGA ≥28 weeks and birth weight ≥800g,

^b^delivery related death,

^c^acute pulmonary embolus G5P4 AROM at 5 cm;

^d^live born;

^e^ p-value from Chi-squared test comparison of proportions from the year 2008 and the year 2015.

#### Qualitative evaluation of the SBA curriculum by senior SBAs

Three major themes emerged, Confidence [in asking questions], Competence [knowing how to do it], and Teamwork [working together] from semi-structured interviews of local SBAs who had taught at SMRU both before and after the SBA curriculum was introduced.

All interviewees mentioned increased confidence observed in SBA graduates and their ability to ask questions and how this was a change from the years prior to formal training. Clarifying when you are not sure of something is crucial for safe practice as a SBA, but asking is remarkable in this population because of cultural morés of unquestioning respect for teachers and superiors.

‘Tharamu^#^, I see the student…at first they are struggling then they are confident. If I compare them [SBA before the training program and SBA after the training program] they are more confident.” [She qualified her observations by talking about the importance of continued supervision]; ‘But Tharamu they are only confident if they have someone to stand by them.’

SBA 2, SBA for 19 years.

The second theme to emerge was, Competence [knowing how to do it], reflecting the experienced SBA’s impression that new SBA graduates were able to give maternity care safely with minimal supervision in contrast to the situation prior to the introduction of formal training.

The SBAs qualified their mostly positive responses with observations that not all students were achieving highly.

‘[Yes it is] good Tharamu^#^, students know all step-by step [meaning clinical protocols], one or two go slowly, slowly….some make mistakes over again but they learn when we show [tell] them’.

SBA 4, SBA for 26 years.

‘After midwife* training … [the] students can do all now [she paused] but …, emergencies not OK -PPH not OK yet, but [students and new graduate SBAs] OK for… OK for observations [routine care] and normal deliveries but we [supervising SBAs] need to supervise for emergencies.’

SBA 3, SBA for 20 years.

The third theme of Teamwork emerged from the experienced SBA’s reported observations of student’s ability to work effectively in teams, particularly during emergencies.

‘Tharamu^#^, before training…we rush a lot and for example PPH [postpartum haemorrhage] we rush a lot and nobody is sure [what to do]-we don’t have close observations …but now we know how to do [observe clinical condition following PPH].’

SBA 3, SBA for 20 years.

Students were often reluctant to ask questions and were sometimes delayed in their attainment of problem solving and critical thinking skills in both classroom and clinical settings. Adult learning methodologies were encouraged during classroom and clinical teaching sessions and senior SBAs have commented on the changes;

‘Tharamu^#^ they are good midwives*, they want to know, they ask…many questions [she paused and then started laughing] and sometimes we don’t know the answers.’

SBA 5, SBA for 7 years.

^#^ Tharamu is a Karen word for teacher or respected female.

* At SMRU, SBAs are called midwives

### Qualitative evaluation of the TOT program by participants

Three of the five teachers were available for participation in semi-structured interviews and were asked how they felt about teaching and to compare before and after the train-the-trainer program. Three main themes emerging from their responses were similar to the themes arising from the interviews with senior SBAs; Confidence [not being scared], Language [being understood] and Questions [knowing how to ask and answer questions]. They all expressed more confidence in knowing the SBA curriculum content as well as confidence in using adult learning techniques and overcoming their fear of asking and being able to answer questions during teaching sessions. One SBA teacher expressed relief at being able to manage her stress better.

‘[Antenatal care is] easy for me, but other topics I feel very stressed [starts to laugh] and not sleeping before lessons and studying all night before the lesson and worried a lot …and …and *very* scared [she laughed and stressed the word *very* to make her point], but now [it is] easier.’

SBA Teacher 1, SBA for 19 years.

Languages concern was a universal theme in all the interviews as although all SBA teachers speak three or four languages fluently they worry about their proficiency to teach in languages other than their mother tongue and to be able to communicate complex medical terminology.

‘I have to struggle a lot …, [laughing!] last teaching [previous SBA program] I feel very stressed and worried about whether I can speak the correct language to explain [She does not speak fluent Burmese and many students would speak only Burmese, not Karen], but when they pass I feel *very* happy—[she is laughing and stressing the word *very* by singing it!].

SBA Teacher 1, SBA for 19 years.

However it was the topic of questioning techniques that animated the teachers the most and about which they appeared very proud. They expressed increased confidence in being able to ask and answer questions but also confidence in being able to use questioning techniques to assess understanding in students and to open up discussion in the classroom or small groups.

‘Before [TOT] when doctors give lectures they are talking, talking, talking and ask no questions and very boring Tharamu^#^. And no time [for the students] to ask questions. Now I know how to ask questions and make it not boring and make the students happy—that’s why I like teaching now [because I know how] to make the students happy. [When the] students [are] sleeping and not interested now I know how to make it not boring-we can do things, ask questions, show them and give [play] games.’

SBA Teacher 3, SBA for 20 years.

This evaluation demonstrated an understanding of the SBA teacher’s ability to incorporate adult learning methodology into their practice and have the confidence to abandon some previous more didactic methods of teaching.

## Discussion

All students completing SMRU SBA training were competent to provide BEmONC to women and their families living in refugee and migrant clinics along the Thai-Myanmar border. This is important as it is estimated that in low-income countries, universal coverage of midwifery interventions for maternal and newborn health could prevent 61% of maternal, fetal and neonatal deaths [7]. The robustness of this program is validated by SMRU perinatal indicators, which were not adversely affected either during or following SBA programs. Indeed the proportion of women giving birth in SMRU facilities increased over time, due in part to the opening of the Mawker Thai birth unit in April 2010 and this increase may be interpreted as a vote of confidence for the SBA led birth units. Trends in perinatal indicators remain encouraging with the new graduates now forming a majority of the workforce. It is likely that the SBA training has played a role in these improvements, however the data should not be over-interpreted as other contemporaneous changes may have had an influence. Most former evaluations of SBA training have not been able to support clinical skills achievement with perinatal indicators [43–45].

### Theory results

About 50% of pregnant women can read, reflecting poor literacy in this population. Low levels of literacy accompanied by a maximum level of 10^th^ standard education are factors that limit availability of suitable applicants for the SBA program [46]. Previous educational experiences of most SBAs were in didactic environments with limited resources, expectations of rote learning and a culture of unquestioning respect for the teacher. The choice of using MCQ for assessment by was influenced by the difficulty of assessing even short written answers with different levels of literacy in English and the complexity of translation and back translation into several different languages. Written language assessment can be too complex to effectively determine SBA competence in settings where multiple languages and different levels of literacy in any language are common [17]. The mix of spoken languages are part of the daily working environment in clinics along the Thailand-Myanmar border and while most SBA students were fluent in spoken Burmese, their mother tongues were usually one of the local Karen languages and for some, also Thai. Students had mixed ability in speaking, reading or writing in English, however as English is the working language at SMRU, they welcomed any opportunity to increase their English competence and expressed a preference for English-language printed learning materials.

Despite these educational disadvantages, SBAs living in the community they serve have been able to achieve high academic and clinical standards during preparation to become SBAs [47]. As highlighted by others, solutions to post-conflict delivery of BEmONC are contextual and more likely to be successful in NGO run facilities [48, 49] and with inter-country collaboration to increase SBA capacity [14, 50]. This maybe explained in part by the limited bureaucratic obstacles required to implement a curriculum within marginalized populations [51]. The cost of training and up-skilling midwives in low and middle income countries (LMIC) is less than that of up-skilling obstetricians and reduction in maternal and neonatal mortality and morbidity is greatest when obstetricians and SBAs are up-skilled together [11].

### Clinical competency results

The scope of competencies achieved in the accelerated SBA training allows students to achieve WHO SBA requirements and the ICM competencies adapted to suit the local environment (Table 5) [9]. Achievement of clinical competence did not appear to be influenced by each SBA student’s educational background, literacy in English, age or previous work experience, however strong conclusions are precluded given the low statistical power. Of note is the enrolment of three former TBAs in the first SBA training program. These women all successfully completed the program and have become valued SMRU team members and an important community influence for women choosing to have facility births. The supportive framework for achieving clinical competence under supervision of strong mentorship is likely to be one of the main contributing factors to maintenance of the positive direction of the perinatal indicators. Adult learning methodologies were encouraged; for example, small group work, student-led discussion, role-play, simulations, demonstrations, and student presentations, and despite the initial challenge of these being often unfamiliar ways of learning, students were able to achieve remarkable results. Strong mentorship from SBA trainers also had a positive influence on SBA students and helped to overcome some barriers to learning found in a society where cultural morés mitigate against attaining adult learning skills. Despite these constraints, many students in both SBA and TOT programs demonstrated an eagerness to learn, resilience, persistence and robust use of humour to achieve highly in challenging personal circumstances and with very few material resources.

### Retention and attrition

Attrition of graduates was a serious problem and has adversely affected staff numbers and morale in SMRU workplaces. The turnover in SMRU staff has also occurred amongst other cadres of health workers, but the main concern for SMRU was that the attrition of SBAs would reduce the capacity of the unit to provide safe maternity care. Even enthusiastic SBAs face many barriers to further professional learning, including lack of English language proficiency, time constraints, and limited access to courses. These barriers are exacerbated by no access to the Internet, lack of a local support person, and unfamiliarity with adult learning practices or critical analysis of information. In other low-income settings it has been shown that strong leadership and supervision for mid-level health workers is associated with high levels of job satisfaction and reduced intention to leave [52]. Retention of staff living and working in a refugee camp is difficult for many reasons, not least because of the uncertainty of waiting to be relocated to a third country. Reasons for leaving local employment are listed in Table 2. If the trend for Myanmar to transition to a democratic society persists, the continued loss of skilled workers from SMRU refugee and migrant communities may benefit internally displaced Myanmar residents along the Myanmar-Thailand border. For example, we are aware of at least two graduate SBAs who are working as village midwives inside Myanmar.

### Sustainability

Refugee settings were never meant to be sustainable and this uncertainty is one of the reasons there is a paucity of evidenced based programs such as this [43]. It has recently been suggested by others that provision of short-term unregulated SBA courses detracts from quality of care and investment in midwifery training to international standards [53]. However, in this refugee population there is no access to midwifery regulation authorities or educational institutions and therefore no choice. We have shown that it is possible to provide effective training of SBAs in a resource-limited setting by designing a fit-for purpose curriculum, training local staff to be teachers, providing incentives for graduates to remain within their communities and thereby reducing the reliance on expatriate workers. These educational activities require spending of limited resources [11] but overall the risk benefit ratio remains in favour of training, as the potential to reduce maternal-newborn mortality and morbidity until refugee camps are abandoned, is significant [20].

The UN Sustainable Development Goal (SDG) 3c states that to; *Ensure healthy lives and promote wellbeing for all at all ages [countries must] substantially increase health financing and the recruitment*, *development*, *training and retention of the health workforce in developing countries*, *especially in least developed countries and small island developing states* [54]. Experience in this and other resource poor settings has shown that to achieve quality SBA or midwifery workforce training, collaboration with higher income countries to fund and share expertise was valuable [14, 50].

### Strengths and limitations

This study adds important evidence to the literature on training of SBAs in resource-poor settings. Strengths of this evaluation include the use of multiple methods of program evaluation to demonstrate that in neglected populations it is possible to provide safe and sustainable training for SBAs as reflected in the good perinatal outcomes in this setting [55]. Programs in other resource poor-settings have focussed on fewer component evaluations [56]. Reliability in education increases with multiple evaluation methods [57] and this report is unique in being able to present various methods. Within our study we used extensive process evaluation, combined with qualitative interviews and capacity to monitor perinatal outcomes using routine data. This last point is important because the aim of health education is to acquire competencies and skills that promote safe clinical practice, not only in simulation but also in real life [58]. The multiple methods of program evaluation reported in this manuscript extend observations about what can be achieved when necessary, as this program arose from dire need.

Qualitative analysis conducted as a routine part of course evaluation has allowed for a more nuanced evaluation of the program from the perspective of both supervising SBA staff and SBA teachers. A recognized limitation is the lack of significant input from SBA students in qualitative program evaluation and to provide a more comprehensive assessment of continued competence [37]. However, experience in other low-resource settings with SBAs has demonstrated successful achievement of competence in health workers with low levels of education [59]. The SMRU training program took place under relatively stable conditions with established birthing units and so may not be common to other neglected or marginalized populations. Nevertheless this was a setting where most women birthed at home just 20 years ago [30]. A secondary limitation is lack of an analysis of cost effectiveness of the program [14].

## Conclusions

This evaluation has shown that in a neglected setting, local women can be trained to become SBAs to provide effective care. Although our results may not be applicable to other similar settings, our findings contribute to an understanding of how an accelerated SBA training can ensure safe maternity care where qualified midwives are unavailable. In neglected settings, education needs to be tailored to local needs. These conclusions would be strengthened by further research in similar settings. Importantly, we show that an initial education program can be sustained by local teachers, when they are given adequate training, support and an enabling environment.

## Supporting Information

S1 Fig Map of SMRU clinics. (TIF)

S1 File SBA clinical practice logbook. (PDF)
