# Supplementary figures and images for: Accelerated Training of Skilled Birth Attendants in a Marginalized Population on the Thai-Myanmar Border: A Multiple Methods Program Evaluation
Source: PLoS One. 2016 Oct 6;11(10):e0164363. doi: 10.1371/journal.pone.0164363 (PMC5053505; doi:10.1371/journal.pone.0164363)

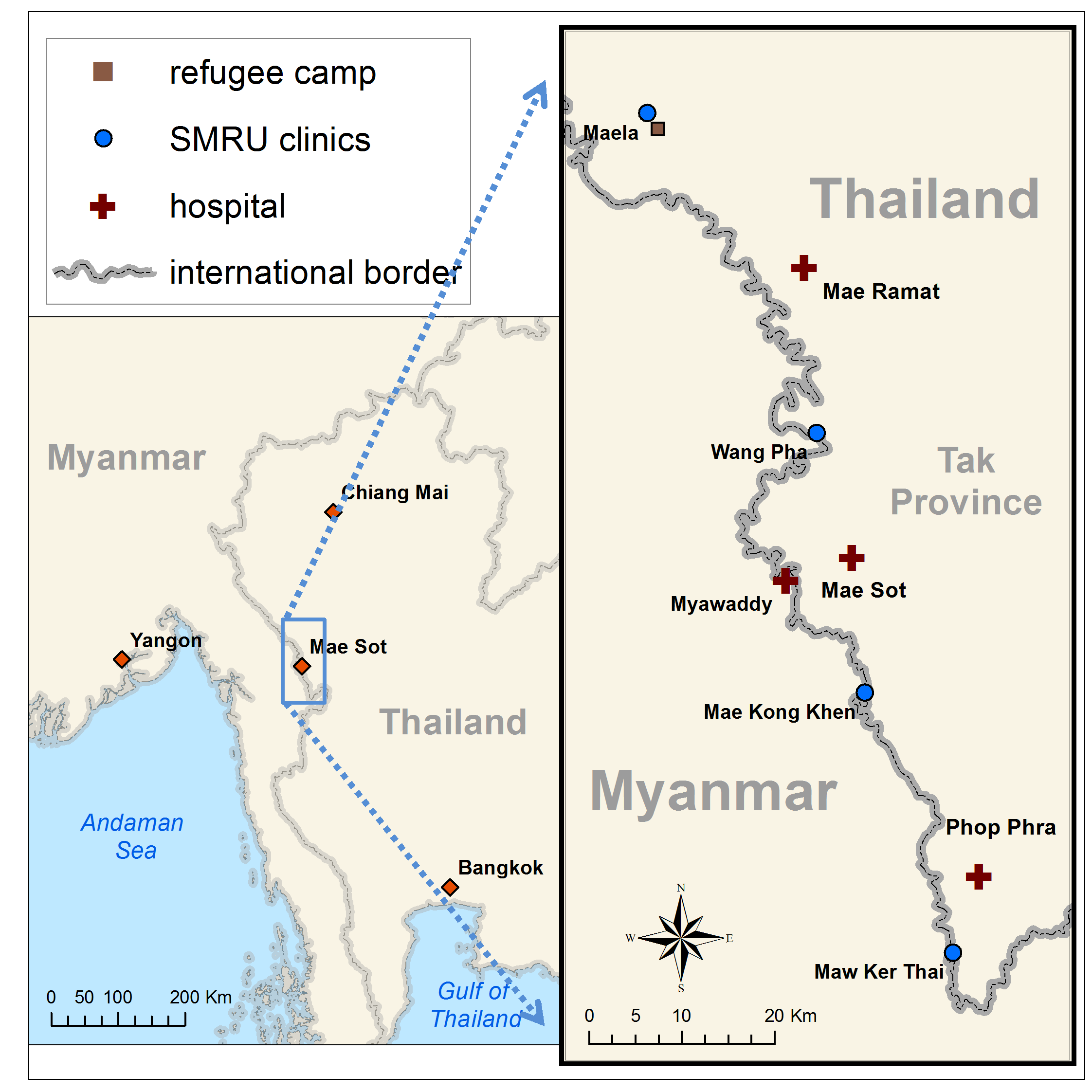

Supplement: S1 Fig — (TIF) [file pone.0164363.s001.tif]
