# Supplementary material for: Accelerated Training of Skilled Birth Attendants in a Marginalized Population on the Thai-Myanmar Border: A Multiple Methods Program Evaluation
Source: PLoS One. 2016 Oct 6;11(10):e0164363. doi: 10.1371/journal.pone.0164363 (PMC5053505; doi:10.1371/journal.pone.0164363)
Supplement: S1 File — (PDF) [file pone.0164363.s002.pdf]

Midwifery Clinical Practice Log Book

SMRU Midwifery Training Program 2010-2011

Student Name..

BRING THIS BOOK TO WORK EVERY DAY.

Table of contents

Introduction to Log Book. 3

Antenatal care (ANC) instructions. 4

Table 1. First visit or new pregnancy. 6

Table 2. PMTCT counselling and consent for testing. 8

Table 3. Antenatal care and belly check. 10

Table 4. Observe ultrasound scan (US) in pregnancy. 19

Table 5. Complicated (abnormal) pregnancy. 21

Labour and delivery care instructions. 26

Table 6. Care in labour (not delivery). 29

Table 7. Vaginal examination for Bishop score and/or membrane sweep. 31

Table 8. Vaginal examination in labour. 32

Table 9. Speculum vaginal examination. 36

Table 10. Normal delivery (NVD). 37

Table 11. Reception of the newborn. 42

Table 12. Observe cutting of episiotomy. 44

Table 13. Observe repair of the perineum. 45

Table 14. Complications (problems) of labour and delivery. 46

Postnatal care (PNC) instructions. 53

Table 15. Postnatal daily care of mother and baby. 57

Table 16. Postnatal complications (problems) of mother or baby. 62

Table 17. Family planning advice. 67

Table 18. Handover to doctor medic or senior midwife (Case presentation). 69

Sick newborn babies instructions. 71

Table 19. Observe care of babies in SCBU or IPD. 72

Clinical skills checklists instructions. 74

Midwifery Clinical Skill 1. ANC first visit or new pregnancy. 75

Midwifery Clinical Skill 2. Belly check. (Abdominal examination). 77

Midwifery Clinical Skill 3. Vaginal examination for Bishop’s score or membranes sweep. 79

Midwifery Clinical Skill 4. Vaginal examination in labour. 81

Midwifery Clinical Skill 5. Speculum vaginal examination. 83

Midwifery Clinical Skill 6. Normal delivery (NVD). 84

Midwifery Clinical Skill 7. Reception of the newborn. 86

Midwifery Clinical Skill 8. Resuscitation of the newborn. (Role play). 88

Midwifery Clinical Skill 9. Postnatal discharge (DC). 90

Midwifery Clinical Skill 10. Handover of cases to doctor, medic or senior midwife. 92

Reflection on practice instructions. 93

Case study instructions. 95

Introduction to Log Book.

- This is your book to write down everything you do during midwifery training.
- Write your name on the logbook and keep it safe.
- **KEEP THIS BOOK WITH YOU ALL THE TIME.**
- It will be part of the examinations to pass midwifery at SMRU.
- Ask the supervisor (senior midwife, midwife teacher or doctor) to sign your logbook every day.
- If you lose your book you must tell the supervisor straight away.
- If you need more pages for the logbook, ask the midwife teacher.
- If you are not sure about how to do anything, ASK FOR HELP before starting.

Antenatal care (ANC) instructions.

(Table 1.) First visit or new pregnancy.

- Do x 3 first visits with the supervisor explaining (more if needed) before you do any by yourself.
- Then do x 3 with the supervisor double checking. **Now do the first assessment.** If "safe", you can continue. **If "not safe", you must see the midwife teacher before you continue.**
- **Then do x 3 more with supervisor double checking before doing repeat assessment.**
- **When doing by yourself, ask the supervisor to check every time.**

When you have finished ≥ 10 first visits and can do everything with no mistakes, then you can ask supervisor to assess you for Clinical Skill 1. First visit or new pregnancy.

<u>For every first visit;</u>

- Take a full history.
- Calculate EDD using woman says and Ultra-sound (US).
- Observe when the woman goes to the medic for physical examination, write down what you see.
- Write down on ANC card, weight, EGA, FH, FHB, BP, T, HCT, MS, anemia treatment.
- Do full belly check, if >12 weeks EGA. If abnormal, show to senior midwife.
- Explain to the woman danger signs; come back to SMRU for ANC and delivery.

(Table 2.) PMTCT counselling and consent for testing. See table for instructions.

(Table 3.) Antenatal care and belly check.

- Do x 3 belly checks with the supervisor explaining (more if needed) before you do any by yourself.
- Then do x 3 with the supervisor double checking. **Now do the first assessment.** If "safe", you can continue. **If "not safe", you must see the midwife teacher before you continue.**
- **Then do x 3 with supervisor double checking before doing repeat assessment.**
- **When doing by yourself, if not sure ALWAYS ASK FOR HELP.**
- **Write down** belly check and examination of **at least 50 women**:

When you have finished ≥ 30 and can do belly check with no mistakes, ask the supervisor to assess you for Clinical Skill 2. Belly check.

Also do and write down.

- EGA, fundal height, (≥ 28 weeks EGA write down fetal lie, presentation, position, engagement.)
- Fetal heart beat (FHB), maternal weight, temperature, BP, HCT, malaria screen,
- Tetanus vaccination, any medical treatment, nutritional advice, supplements and any food rations.
- **Education** about ANC visits, delivery at SMRU, minor disorders of pregnancy, signs of labour, danger signs, when to come to SMRU.

(Table 4.)Observe ultrasound scan (US) in pregnancy.

- When you are on ANC duties, you will watch in ultrasound for 1-2 days.
- Write down at least 10 scans. Try to see scans from each trimester of pregnancy.

(Table 5.) Complicated pregnancy.

- If you look after a woman with complicated (abnormal) pregnancy, write it down **At least 50.**
- If you helped with treatment, write down what you did.

Table 1. First visit or new pregnancy.				
**Date, ANC code.**	**Calculate EDD mother says ;EGA by US Gravida, Parity.**	**Any Risk factors. Any medical problems. education for danger signs, nutrition, deliver at SMRU.**	**Feedback from supervisor**	**Sign.**
**1**			**Watch first visit. Supervisor must explain step by step**	
**2**			**Watch first visit. Supervisor must explain step by step**	
**3**			**Watch first visit. Supervisor must explain step by step**	
**4**			**Do yourself. Supervisor watches you and gives feedback**	
**5**			**Do yourself. Supervisor watches you and gives feedback**	
**6**			**Do yourself. Supervisor watches you and gives feedback First assessment.**	
**7**				
**8**				
**9**				
**10**				

Table 1. First visit or new pregnancy.				
**Date, ANC code.**	**Calculate EDD mother says ;EGA by US Gravida, Parity.**	**Any Risk factors. Any medical problems. education for danger signs, nutrition, deliver at SMRU.**	**Feedback from supervisor**	**Sign.**
**11**				
**12**				
**13**				
**14**				
**15**				
**16**				
**17**				
**18**				
**19**				
**20**				

Add more pages if you need.

Table 2. PMTCT counselling and consent for testing.		
**Instructions.**<br>• When you are working in ANC, you will go to PMTCT counselling for one day.<br>• The PMTCT counsellor will do pre-test and post-test counselling<br>• You will watch, but not do this by yourself.<br>• Write down here what you observed, any tests and any outcomes.<br>• **Remember what you hear and what you see at PMTCT is confidential.**<br>• **Do not discuss with anyone outside the room.**		
**1. PMTCT observation. Pre-test.**	**Write down** what happened, any tests and any outcomes	**Sign**
**2. PMTCT observation. Post-test.**	**Write down** what happened, any tests and any outcomes	**Sign.**
**3. PMTCT observation. Pre-test.**	**Write down** what happened, any tests and any outcomes	**Sign.**

**4. PMTCT observation. Post-test.**	**Write down** what happened, any tests and any outcomes	**Sign.**

Add more pages if you need.

Table 3. Antenatal care and belly check.

Date, ANC code.	EGA	Weight (Kg)	Fundal Height (FH)	Fetal position	FHB	BP	Temp	HCT	MS	Any abnormalities, advice, education. Feedback from supervisor.	Sign.
1										**Watch supervisor do belly check.** Supervisor must explain step by step	
2										**Watch supervisor do belly check.** Supervisor must explain step by step	
3										**Watch supervisor do belly check.** Supervisor must explain step by step	
4										DO yourself. Supervisor to check and give feedback	
5										DO yourself. Supervisor to check and give feedback	
6										DO yourself. Supervisor to check and give feedback. First Assessment	
7											
8											
9											
10											

Table 3. Antenatal care and belly check.											
**Date, ANC code.**	**EGA**	**Weight (Kg)**	**Fundal Height (FH)**	**Fetal position**	**FHB**	**BP**	**Temp**	**HCT**	**MS**	**Any Abnormalities, Advice, Education. Feedback from supervisor.**	**Sign.**
**11**											
**12**											
**13**											
**14**											
**15**											
**16**											
**17**											
**18**											
**19**											
**20**											

Table 3. Antenatal care and belly check.											
**Date, ANC code.**	**EGA**	**Weight (Kg)**	**Fundal Height (FH)**	**Fetal position**	**FHB**	**BP**	**Temp**	**HCT**	**MS**	**Any Abnormalities, Advice, Education. Feedback from supervisor.**	**Sign.**
**21**											
**22**											
**23**											
**24**											
**25**											
**26**											
**27**											
**28**											
**29**											
**30**											

Table 3. Antenatal care and belly check.											
**Date, ANC code.**	**EGA**	**Weight (Kg)**	**Fundal Height (FH)**	**Fetal position**	**FHB**	**BP**	**Temp**	**HCT**	**MS**	**Any Abnormalities, Advice, Education. Feedback from supervisor.**	**Sign.**
**31**											
**32**											
**33**											
**34**											
**35**											
**36**											
**37**											
**38**											
**39**											
**40**											

Table 3. Antenatal care and belly check.											
**Date, ANC code.**	**EGA**	**Weight (Kg)**	**Fundal Height (FH)**	**Fetal position**	**FHB**	**BP**	**Temp**	**HCT**	**MS**	**Any Abnormalities, Advice, Education. Feedback from supervisor.**	**Sign.**
**41**											
**42**											
**43**											
**44**											
**45**											
**46**											
**47**											
**48**											
**49**											
**50**											

.

Table 3. Antenatal care and belly check.											
**Date, ANC code.**	**EGA**	**Weight (Kg)**	**Fundal Height (FH)**	**Fetal position**	**FHB**	**BP**	**Temp**	**HCT**	**MS**	**Any Abnormalities, Advice, Education. Feedback from supervisor.**	**Sign.**
**51**											
**52**											
**53**											
**54**											
**55**											
**56**											
**57**											
**58**											
**59**											
**60**											

Add more pages if you need

Table 4. Observe ultrasound scan (US) in pregnancy.			
**Date**<br>**ANC code.**	**Reason for scan**	**Results of scan and EGA. Any treatment or advice to woman.**	**Sign.**
1			
2			
3			
4			
5			
6			
7			
8			
9			
10			

Table 4. Observe ultrasound scan (US) in pregnancy.			
**Date**<br>**ANC code.**	**Reason for scan**	**Results of scan and EGA. Any treatment or advice to woman.**	**Sign.**
11			
12			
13			
14			
15			
16			
17			
18			
19			
20			
21			
22			

Please add more pages if needed

Table 5. Complicated (abnormal) pregnancy.					
**Date ANC code.**	**EGA.**	**Complication.(may be more than one, e.g. abortion and infection)**	**Any treatment, advice or education**	**Admission or follow-up**	**Sign.**
1		Abortion			
2		Abortion			
3		Abortion			
4		Abortion			
5		Abortion			
6		Abortion			
7		Abortion			
8		Abortion			
9		Abortion			
10		Abortion			

Table 5. Complicated (abnormal) pregnancy.					
**Date ANC code.**	**EGA.**	**Complication.(may be more than one, e.g. abortion and infection)**	**Any treatment, advice or education**	**Admission or follow-up**	**Sign.**
11		Anaemia			
12		Anaemia			
13		Anaemia			
14		Anaemia			
15		Anaemia			
16		Anaemia			
17		Anaemia			
18		Anaemia			
19		Anaemia			
20		Anaemia			
21		Anaemia			

Table 5. Complicated (abnormal) pregnancy.					
**Date ANC code.**	**EGA.**	**Complication in Pregnancy.**	**Any treatment, advice or education**	**Admission or follow-up**	**Sign.**
22		High BP / pre-eclampsia			
23		High BP / pre-eclampsia			
24		High BP / pre-eclampsia			
25		High BP / pre-eclampsia			
26		High BP / pre-eclampsia			
27		High BP / pre-eclampsia			
28		APH			
29		APH			
30		APH			
31		APH			
32		APH			

Table 5. Complicated (abnormal) pregnancy.					
**Date ANC code.**	**EGA.**	**Complication in Pregnancy.**	**Any treatment, advice or education**	**Admission or follow-up**	**Sign.**
33		Pre Term Labour (PTL)			
34		Pre Term Labour (PTL)			
35		Pre Term Labour (PTL)			
36		Pre Term Labour (PTL)			
37		Urinary Tract Infection (UTI)			
38		Urinary Tract Infection (UTI)			
39		Urinary Tract Infection (UTI)			
40		Urinary Tract Infection (UTI)			
41		Urinary Tract Infection (UTI)			
42		Infection			
43		Infection			

Table 5. Complicated (abnormal) pregnancy.					
**Date ANC code.**	**EGA.**	**Complication in Pregnancy. (write down)**	**Any treatment, advice or education**	**Admission or follow-up**	**Sign.**
44		Malaria			
45		Malaria			
46		Malaria			
47		Malaria			
48					
49					
50					
51					
52					
53					
54					

Add more pages if you need

Labour and delivery care instructions.

(Table 6.) Care in Labour.

- Write down every time you look after a woman in labour for more than 2 hours, even if you do not see or do the delivery.

(Table 7.) Vaginal examination for Bishop's score ,

- Do x 3 with the supervisor explaining (more if needed) before you do any by yourself.
- Then do x 3 with the supervisor double checking. **Now do the first assessment.** If "safe", you can continue. **If "not safe", you must see the midwife teacher before you continue.**
- **Then do x 3 with supervisor double checking before doing repeat assessment.**
- You must do at least 13 VE for Bishop's score.

(Table 8.) Vaginal examinations in labour;

- Do x 3 with the supervisor explaining (more if needed) before you do any by yourself.
- Then do x 3 with the supervisor double checking. **Now do the first assessment.** If "safe", you can continue. **If "not safe", you must see the midwife teacher before you continue.**
- **Then do x 3 with supervisor double checking before doing repeat assessment.**
- You must do at least 33 VE in labour.

(Table 9.) Vaginal examinations for speculum

- Do x 3 with the supervisor explaining (more if needed) before you do any by yourself.
- Then do x 3 with the supervisor double checking. **Now do the first assessment.** If "safe", you can continue. **If "not safe" you must see the midwife teacher before you do anymore.**
- **Then do x 3 with supervisor double checking before doing repeat assessment.**
- You must do at least 13 VE for speculum.

When you can each vaginal examination with no mistakes, ask the supervisor to assess you for Clinical Skills 4, 5, 6.

(Table 10.) Normal delivery* (NVD). Must do **at least 26**.

**delivery of twins can be one normal delivery if they are head/head presentation.*

- **Watch 3 deliveries at SMRU before you do any with your hands touching.**
- **Do x 3 deliveries (more if needed) with the senior midwife standing next to you with gloves on and hands touching, BEFORE you do your first assessment.**
- **Now do the first assessment.** If "safe", you can continue. **If "not safe" you must see the midwife teacher and do another x 3 deliveries with the senior midwife standing next to you with gloves on and hands touching <u>before</u> doing repeat assessment.**
- **The supervisor will still be watching every NVD until you have done your final assessment.**
- **When you have finished at least 26 deliveries and can do everything safely and with no mistakes, then you can ask supervisor to assess you for Clinical Skill 3. Normal delivery.**
-

<u>(Table 11.) Reception of the newborn. (Cut the cord)</u>

- **Watch x 3 before you do any yourself**.
- Then do x 3 with the senior midwife standing next to you with gloves on and hands touching.
- **Now do the first assessment.** If "safe", you can continue. **If "not safe" you must see the midwife teacher and do another x 3 with the supervisor standing next to you with gloves on and hands touching <u>before</u> doing repeat assessment.**
- **When you have done at least 26 newborn receptions and can do everything with no mistakes, ask the supervisor to do your final assessment for Clinical Skill 7. Reception of the newborn.**

(Table 12.) Observe cutting of episiotomy. (You may not be able to see this because episiotomy is rare).

- Watch the senior midwife, teacher or doctor do x 5
- Do x 5 only with senior midwife, teacher or doctor with gloves on helping.

(Table 13.) Observe Suturing of the perineum

- Watch the senior midwife or doctor do perineal suturing and write down.
- **DO NOT DO ANY SUTURING of the perineum unless**
    - **You have already finished lectures/workshop on how to do perineal suturing**
    - **You have permission from the doctor at your clinic**
    - **You have watched x 5 with explanation.**
    - **The supervisor has gloves on and is helping for x 5.**
    -

(Table 14.) Complications (problems) of labour and delivery

- If the woman you are looking after has complications during labour or delivery, write down her treatment, any drugs and what happened.
- If you do anything to help with her treatment or care, write it down

Resuscitation of the newborn.

- When you have done the lecture on newborn resuscitation and practiced many times (role play) with no mistakes, the supervisor will assess you for **Clinical Skill 7. Resuscitation of the newborn.**

Table 6. Care in labour (not delivery).

Date<br>ANC code.	Parity<br>EGA	Belly<br>Check	Any risk factors problems or<br>complications.	What went well? What did I learn?<br>What to do better next time?	Sign.
1					
2					
3					
4					
5					
6					
7					
8					
9					
10					

Table 6. Care in Labour (not delivery).					
**Date**<br>**ANC code.**	**Parity**<br>**EGA**	**Belly**<br>**Check.**	**Any risk factors problems or**<br>**complications.**	**What went well? What did I learn?**<br>**What to do better next time?**	**Sign.**
11					
12					
13					
14					
15					
16					
17					
18					
19					
20					

Add more pages if you need

Table 7. Vaginal examination for Bishop score and/or membrane sweep.

Date, ANC code.	Parity EGA.	Reason for VE.	Belly Check FHB.	Bishop Score And results	Any problems, plan for care. Feedback from senior midwife	Sign.
**1**					**Checked and explained by supervisor**	
**2**					**Checked and explained by supervisor**	
**3**					**Checked and explained by supervisor**	
**4**					**Supervisor to double check**	
**5**					**Supervisor to double check**	
**6**					**Supervisor to double check**<br>**First Assessment**	
**7**						
**8**						
**9**						
**10**						
**11**						
**12**						
**13**						
**14**						
**15**						

Add more pages if you need

Table 8. Vaginal examination in labour.

Date, ANC code.	Parity EGA.	Reason for VE.	Belly Check FHB.	Results	Any problems, plan for care. Feedback from senior midwife	Sign.
1					Checked and explained by supervisor	
2					Checked and explained by supervisor	
3					Checked and explained by supervisor	
4					Supervisor to double check	
5					Supervisor to double check	
6					Supervisor to double check<br>First assessment	
7						
8						
9						
10						
11						
12						
13						
14						
15						

Table 8. Vaginal examination in labour.						
**Date, ANC code.**	**Parity EGA.**	**Reason for VE.**	**Belly Check FHB**	**Results**	**Any problems, plan for care. Feedback from senior midwife**	**Sign.**
**16**						
**17**						
**18**						
**19**						
**20**						
**21**						
**22**						
**23**						
**24**						
**25**						
**11**						
**26**						
**27**						
**28**						
**29**						
**30**						

Table 8. Vaginal examination in labour.						
**Date, ANC code.**	**Parity EGA.**	**Reason for VE.**	**Belly Check FHB**	**Results**	**Any problems, plan for care. Feedback from senior midwife**	**Sign.**
**31**						
**32**						
**33**						
**34**						
**35**						
**36**						
**37**						
**38**						
**39**						
**40**						
**41**						
**42**						
**43**						
**44**						
**45**						
**50**						

Table 8. Vaginal examination in labour.						
**Date,**<br>**ANC code.**	**Parity**<br>**EGA.**	**Reason**<br>**for VE.**	**Belly Check**<br>**FHB**	**Results**	**Any problems, plan for care.**<br>**Feedback from senior midwife**	**Sign.**
**51**						
**52**						
**53**						
**54**						
**55**						
**56**						
**57**						
**58**						
**59**						
**60**						
**61**						
**62**						
**63**						
**64**						
**65**						
**66**						

Add more pages if you need

Table 9. Speculum vaginal examination.					
**Date, ANC code.**	**Parity EGA.**	**Reason for Speculum VE.**	**Results**	**Any problems, plan for care. Feedback from senior midwife/doctor**	**Sign.**
**1**				**Checked and explained by supervisor**	
**2**				**Checked and explained by supervisor**	
**3**				**Checked and explained by supervisor**	
**4**				**Supervisor to double check if needed**	
**5**				**Supervisor to double check if needed**	
**6**				**Supervisor to double check if needed** **First assessment**	
**7**					
**8**					
**9**					
**10**					
**11**					
**12**					
**13**					
**14**					
**15**					
**16**					

Add more pages if you need

Table 10. Normal delivery (NVD).				
**Date, ANC Code**	**Parity EGA, HCT.**	**Belly check FHB.**	**MUST WATCH X3 BEFORE DOING ANY-SEE TABLE 6, Watch Normal Delivery. Write down what happened during delivery. Any problems during delivery. Feedback from senior midwife, teacher or doctor (supervisor)**	**Sign.**
**1**			**Watch NVD. Supervisor must explain delivery step by step.**	
**2**			**Watch NVD. Supervisor must explain delivery step by step.**	
**3**			**Watch NVD. Supervisor must explain delivery step by step.**	
**4**			**Do NVD. Supervisor must help with gloves on and hands touching.**	
**5**			**Do NVD. Supervisor must help with gloves on and hands touching.**	
**6**			**Do NVD. Supervisor must help with gloves on and hands touching.**<br>**Do FIRST ASSESSMENT**	
**7**				
**8**				
**9**				

Table 10. Normal delivery.				
**Date,**<br>**ANC Code**	**Parity**<br>**EGA, HCT.**	**Belly check FHB.**	**Write down what happened during delivery. Any problems during delivery**<br>**Feedback from senior midwife/doctor**	**Sign.**
**10**				
**11**				
**12**				
**13**				
**14**				
**15**				
**16**				
**17**				
**18**				

Table 10. Normal delivery.				
**Date,**<br>**ANC Code**	**Parity**<br>**EGA, HCT.**	**Belly**<br>**check**<br>**FHB.**	**Write down what happened during delivery. Any problems during delivery**<br>**Feedback from senior midwife/doctor**	**Sign.**
**19**				
**20**				
**21**				
**22**				
**23**				
**24**				
**25**				
**26**				
**27**				

Table 10. Normal delivery.				
**Date,**<br>**ANC Code**	**Parity**<br>**EGA, HCT.**	**Belly check, FHB.**	**Write down what happened during delivery. Any problems during delivery**<br>**Feedback from senior midwife/doctor**	**Sign.**
**28**				
**29**				
**30**				
**31**				
**32**				
**33**				
**34**				
**35**				
**36**				

Table 10. Normal delivery.				
**Date,<br>ANC Code**	**Parity<br>EGA, HCT.**	**Belly<br>check<br>FHB.**	**Write down what happened during delivery. Any problems during delivery<br>Feedback from senior midwife/doctor**	**Sign.**
**37**				
**38**				
**39**				
**40**				
**41**				
**42**				
**43**				
**44**				
**45**				

Add more pages if you need

Table 11. Reception of the newborn.

Date, ANC code.	Type of delivery EGA	Apgar score. Any resuscitation	Any comments Feedback from senior midwife, teacher or doctor (supervisor)	Sign.
1			Watch. Supervisor must explain step by step.	
2			Watch. Supervisor must explain step by step.	
3			Watch. Supervisor must explain step by step.	
4			Do Reception. Supervisor must help with gloves on and hands touching	
5			Supervisor must help with gloves on and hands touching	
6			Supervisor must help with gloves on and hands touching FIRST ASSESSMENT	
7				
8				
9				
10				
11				
12				
13				
14				

Table 11 Reception of the newborn.				
**Date,**<br>**ANC code.**	**Type of delivery EGA**	**Apgar score. Any resuscitation**	**Any comments**<br>**Feedback from senior midwife or supervisor**	**Sign.**
**15**				
**16**				
**17**				
**18**				
**19**				
**20**				
**21**				
**22**				
**23**				
**24**				
**31**				
**25**				
**26**				
**27**				
**28**				
**29**				
**30**				
**31**				
**32**				
**33**				

Please add more pages if needed

Table 12. Observe cutting of episiotomy.			
**Date and ANC Code.**	**Reason for Episiotomy**	**Write down any problems. What did you learn? Any feedback**	**Sign.**
1.		Watch senior midwife or doctor	
2.		Watch senior midwife or doctor	
3.		Watch senior midwife or doctor	
4.		Watch senior midwife or doctor	
5.		Watch senior midwife or doctor	
6.		Do your self Supervisor must have gloves on and helping	
7.		Do your self. Supervisor must have gloves on and helping	
8.		Do your self. Supervisor must have gloves on and helping	
9.		Do your self. Supervisor must have gloves on and helping	
10.		Do your self. Supervisor must have gloves on and helping	
11.			
12.			
13.			
14.			

Please add more pages if needed

Table 13. Observe repair of the perineum.			
**Date and ANC Code.**	**Tear or episiotomy. Degree of tear.**	**Write down any problems during suturing. What did I learn?**	**Sign.**
1.		Watch senior midwife or doctor	
2.		Watch senior midwife or doctor	
3.		Watch senior midwife or doctor	
4.		Watch senior midwife or doctor	
5.		Watch senior midwife or doctor	
6.			
7			
8			
9			
10			
11			
12			

Please add more pages if needed

Table 14. Complications (problems) of labour and delivery.

**Date, ANC code, EGA Complication (problem)**	**What happened? What treatment was given?**	**Outcomes Why did this happen? Any feedback from supervisor**	**Sign.**
Post term			
Post term			
Post term			
Induction of labour			
Induction of labour			
Augmentation of labour.			
Augmentation of labour			
Twin delivery			
Breech delivery			

Table 14 Complications (problems) of labour and delivery.			
**Date, ANC code, EGA Complication (problem)**	**What happened? What treatment was given?**	**Outcomes Why did this happen? Any feedback from supervisor**	**Sign.**
Breech delivery			
Breech delivery			
Breech delivery			
Vacuum delivery			
Vacuum delivery			
Vacuum delivery			
Forceps delivery			
Antepartum Haemorrhage (APH)			
Antepartum Haemorrhage (APH)			

Preterm Labour			

Table 14 Complications (problems) of labour and delivery.

**Date, ANC code, EGA Complication (problem)**	**What happened? What treatment was given?**	**Outcomes Why did this happen? Any feedback from supervisor**	**Sign.**
Preterm Labour			
Preterm Labour			
Preterm Labour			
Preterm Labour			
Severe Pre-eclampsia			
Severe Pre-eclampsia			
Severe Pre-eclampsia			
Severe Pre-eclampsia			

Severe Pre-eclampsia			

Table 14 Complications (problems) of labour and delivery.			
**Date, ANC code, EGA Complication (problem)**	**What happened? What treatment was given?**	**Outcomes Why did this happen? Any feedback from supervisor**	**Sign.**
Shoulder Dystocia			
Cord Prolapse			
Shock (mother)			
Maternal resuscitation			
Neonatal Resuscitation			
Neonatal Resuscitation			
PPH			
PPH			
PPH			

Table 14 Complications (problems) of labour and delivery.			
**Date, ANC code, EGA Complication (problem)**	**What happened? What treatment was given?**	**Outcomes Why did this happen? Any feedback from supervisor**	**Sign.**
PPH			
PPH			
PPH			
Infection			
Infection			
Malaria			
Malaria			

Table 14 Complications (problems) of labour and delivery.			
**Date, ANC code, EGA Complication (problem)**	**What happened? What treatment was given?**	**Outcomes. Why did this happen? Any feedback from supervisor**	**Sign.**

Please add more pages if needed

Postnatal care (PNC) instructions.

(Table 15.) Postnatal daily care of mother and baby.

- **Watch x 3 postnatal checks with the supervisor explaining do before you do any.**
- **Then do x 3 with the supervisor double checking. Now do the first assessment. If "safe", you can continue. If "not safe", you must see the midwife teacher before you continue.**
- **Then do x 3 more with supervisor double checking before doing repeat assessment.**
- If not sure **ALWAYS ASK** for help. A midwife will check each day until you have passed the final assessment.
- **Write down daily examinations of the mother and baby including;**
    - **Mother:** Assessment of uterus, bladder and bowel function, breastfeeding, vital signs, family planning, education about danger signs for mother and baby.
    - **Baby**: Daily assessment of baby, vital signs, skin colour (Kramer's score) feeding, weight, urine, stools, cord care, vaccination, any problems.

When you have done ≥ 20 PNC discharge with no mistakes, ask the senior midwife, teacher or doctor to assess you for Clinical Skill 10. Postnatal discharge.

(Table 16.) Postnatal complications (problems) of mother or baby

Write down

- Postnatal problem (complications) while the woman is in hospital (IPD or PNC) and any midwifery or medical care. If you help with her treatment, write down what you do.
    - Problem examples; difficult delivery, suture of perineum, infection, malaria, anaemia, bad smell, fever, breastfeeding problem, high BP.
- Sometimes there may be more than one problem, write down all. If there is mother AND baby problem-write down both together.
- **Write down any problems for the baby.**
    - Problem examples; abnormal baby, meconium in bag of waters, low birth weight, chest in drawing, O_2 saturations abnormal, weight loss ≥ 10%, jaundice, sepsis, abnormal vital signs, not sucking strong.
- **If you observe or assist during any operation for sterilisation, write down what you did.**
- If mother comes for follow-up after CS or for any reason, write down reason for follow-up and any examination, observations, treatment, education.

(Table 17.) Family planning advice.

- **Watch x 3 with the supervisor explaining**.
- **Then do x 3 with the supervisor watching you and giving feedback.**
- **You will be assessed as part of Clinical Skill 10. Postnatal discharge.**
- Write down every time you give contraception or family planning advice.
- Advice can be after abortion, during pregnancy, after delivery, or at any follow-up
- If you do pre-operation consent form for sterilisation and check list, write down here.
- Always include advice about folate supplements for a future pregnancy.

(Table 18.) Handover of cases to doctor, midwife or medic.

- **Watch x 3 handovers with the supervisor explaining before you do any.**
- **Then do x 3 with the supervisor watching you and giving feedback. Now do the first assessment. If "safe", you can continue. If "not safe", you must see the midwife teacher before you continue.**
- **Then do x 3 more with supervisor double checking before doing repeat assessment.**

When you have done ≥20 handovers on the ward rounds with the doctor with no mistakes ask the doctor or supervisor to assess you for, Clinical Skill 10. Handover of cases.

Table 15. Postnatal daily care of mother and baby.					
**Date, ANC Code. Postnatal Day.**	**Parity EGA.**	**Mother**	**Baby**	**Comments or feedback from supervisor**	**Sign.**
1				Watch midwife	
2				Watch midwife	
3				Watch midwife	
4				**Supervised by midwife**	
5				**Supervised by midwife**	
6				**Supervised by midwife**<br>**First assessment**	

Table 15. Postnatal daily care of mother and baby.					
**Date, ANC Code. Postnatal Day.**	**Parity EGA.**	**Mother**	**Baby**	**Comments or feedback from supervisor**	**Sign.**
7					
8					
9					
10					
11					
12					
13					

Table 15. Postnatal daily care of mother and baby.					
**Date, ANC Code. Postnatal Day.**	**Parity EGA.**	**Mother**	**Baby**	**Comments or feedback from supervisor**	**Sign.**
14					
15					
16					
17					
18					
19					
20					

Table 15. Postnatal daily care of mother and baby.					
**Date, ANC Code. Postnatal Day.**	**Parity EGA.**	**Mother**	**Baby**	**Comments or feedback from supervisor**	**Sign.**
21					
22					
23					
24					
25					
26					
27					

Table 15. Postnatal daily care of mother and baby.					
**Date, ANC Code. Postnatal Day.**	**Parity EGA.**	**Mother**	**Baby**	**Comments or feedback from supervisor**	**Sign .**
28					
29					
30					
31					
32					
33					
34					

Please add more pages if needed

Table 16. Postnatal complications (problems) of mother or baby.					
**Date, ANC Code. Postnatal Day.**	**Parity EGA.**	**Mother**	**Baby**	**Comments or feedback from supervisor**	**Sign.**
1					
2					
3					
4					
5					
6					

Table 16. Postnatal complications (problems) of mother or baby.					
**Date, ANC Code. Postnatal Day.**	**Parity EGA.**	**Mother**	**Baby**	**Comments or feedback from supervisor**	**Sign.**
**7**					
**8**					
**9**					
**10**					
**11**					
**12**					
**13**					
**14**					
**15**					

Table 16. Postnatal complications (problems) of mother or baby.					
**Date, ANC Code. Postnatal Day.**	**Parity EGA.**	**Mother**	**Baby**	**Comments or feedback from supervisor**	**Sign.**
**16**					
**17**					
**18**					
**19**					
**20**					
**21**					
**22**					
**23**					
**24**					

Table 16. Postnatal complications (problems) of mother or baby.					
**Date, ANC Code. Postnatal Day.**	**Parity EGA.**	**Mother**	**Baby**	**Comments or feedback from supervisor**	**Sign.**
**25**					
**26**					
**27**					
**28**					
**29**					
**30**					
**31**					
**32**					
**33**					

Table 16. Postnatal complications (problems) of mother or baby.					
**Date, ANC Code. Postnatal Day.**	**Parity EGA.**	**Mother**	**Baby**	**Comments or feedback from supervisor**	**Sign.**
**34**					
**35**					
**36**					
**37**					
**38**					
**39**					
**40**					
**41**					
**42**					

Add more pages if you need here

Table 17. Family planning advice.

Date and ANC Code.	Method of family planning	Contraception advice.	Comments and feedback from supervisor	Sign.
1			Watch midwife	
2			Watch midwife	
3			Watch midwife	
4			Midwife supervises and gives feedback	
5			Midwife supervises and gives feedback	
6			Midwife supervises and gives feedback	
7				
8				
9				
10				
11				

Table 17. Family planning advice.				
**Date and ANC Code.**	**Method of family planning**	**Contraception advice.**	**Comments and feedback from supervisor**	**Sign.**
12				
13				
14				
15				
16				
17				
18				
19				
20				
21				
22				

Add more pages if you need here

Table 18. Handover to doctor medic or senior midwife (Case presentation).				
**Date and ANC Code.**	**Relevant Case details**	**Diagnosis/Plan of care**	**Comments and feedback from supervisor**	**Sign.**
1			Watch midwife	
2			Watch midwife	
3			Watch midwife	
4			Senior Midwife or doctor supervises and gives feedback	
5			Senior Midwife or doctor supervises and gives feedback	
6			Senior Midwife or doctor supervises and gives feedback **First assessment**	
7				
8				
9				

Table 18. Handover to doctor medic or senior midwife (case presentation).				
**Date and ANC Code.**	**Relevant Case details**	**Diagnosis/Plan of care**	**Comments and feedback from supervisor**	**Sign.**
10				
11				
12				
13				
14				
15				
16				
17				
18				
19				
20				

Add more pages if you need here

Sick newborn babies instructions.

(Table 19.) Observe care in Special care baby unit (SCBU) or In-patient department(IPD).

Midwifery students will be observers only in SCBU or for sick babies in IPD.

If you look after a mother and baby and the baby is transferred to SCBU or IPD, find out what happened and write down here. If possible, go with the mother and observe admission of baby.

Always check with the doctor, medic or nurse before visiting SCBU or IPD baby.

If you go to SCBU or IPD,

- Wash your hands before touching anything.
- Ask permission from parents and senior nurse before seeing any baby.
- You can help the mother to express milk or give her PNC care when a midwife is supervising you.
- **You MUST NOT give any treatment to the baby.**

Table 19. Observe care of babies in SCBU or IPD.				
**Date, ANC code, EGA and age of baby.**	**Reason for admission to nursery.**	**Diagnosis and any tests.**	**Summary of treatment and outcome.**	**Sign.**
1	Sepsis.			
2	Sepsis.			
3	Sepsis.			
4	Sepsis.			
5	Sepsis.			
6	Low birth weight.			
7	Low birth weight.			
8	Low birth weight.			
9	Low birth weight.			
10	Low birth weight.			

Table 19. Observe care of babies in SCBU or IPD.				
11	Low birth weight.			
12	Low birth weight.			
13	Jaundice requiring phototherapy			
14	Jaundice requiring phototherapy			
15	Jaundice requiring phototherapy			
16	Respiratory distress			
17	Respiratory distress			
18	Congenital abnormality			
19				
20				
21				

Add more pages if you need here

Clinical skills checklists instructions.

- You must pass all these skills before you can graduate as a midwife at SMRU.
- **The assessment can ONLY be done by the senior midwife, midwife teacher or doctor (supervisor).**
- If you fail you can repeat the assessment as many times as you need.
- If you fail > 2 times, you must ask the midwife teacher for help before doing any more.
- There are **two parts** to each clinical skills assessment.
- 1. **First assessment** to check that you can remember the step by step process with no mistakes and that you are a safe beginner. You must pass the first assessment before you can do any more without close supervision.
- 2. **Final assessment** when you are ready to be a midwife and can do everything with no mistakes.
- **Clinical Skill 8. Resuscitation of newborn, this will be by role play. You can be assessed for this skill when you have finished the lectures on Neonatal Resuscitation and have practiced many times.**
- **You must ask your supervisor when you are ready to do each assessment**.

Midwifery Clinical Skill 1. ANC first visit or new pregnancy.	Yes	NA	No
Be kind to the woman; tell her your name, **Give privacy**.			
Ask simple questions and write down:<br>• social history<br>• medical and surgical history<br>• past obstetric history<br>• any risk factors			
Ask where she will go for delivery. Encourage delivery in SMRU clinic. Tell her to come for ANC every week.			
Check if she has Ultrasound Scan yet and write down EGA.			
Check Fundal Height (FH) and write down.			
Check if fetal heart beat (FHB) and write down.			
**Take Blood Pressure (BP) accurately and write down.**			
Watch medic do full physical check of woman. Write down any problems.			
Can take blood tests for haematocrit (HCT) and malaria smear (MS). Can show which form is used for each test.			
Check Tetanus vaccination card and if needed, give correctly.			
Give the woman vitamin supplements and explain how to take them.			
Give basic healthy eating advice and any food rations.			
**Explain to the woman danger signs and when to come to hospital.**			
Can give simple advice regarding minor disorders in pregnancy.			
Ensure every woman has ANC code written down. Tell her to bring it every visit.			
**Can write down all information correctly on Case Report Form (CRF)**.			
Explain everything to the woman and come back to ANC every week.			
When asked, can state normal range of all observations, examinations, and test results and what to do if not normal. If any problems, can say when to refer.			
Encourage the woman to ask any questions. BE KIND!			
First assessment after x3 with supervision from senior midwife or doctor. *Can do step by step.*<br>***Comments or feedback***	Date	Safe	***Not safe****
Supervisor Name ……………………………… Sign……………………………			
Repeat assessment IF NEEDED after x 6 with supervision from senior midwife or doctor. *Can do step by step.* ***Comments or feedback.***	Date	Safe	***Not safe****
Supervisor Name ……………………………… Sign……………………………			
Final assessment after at least 50 by yourself. Comments or feedback	Date	Pass	Fail
Supervisor Name ……………………………… Sign……………………………			
Repeat final assessment IF NEEDED. Comments or feedback	Date	Pass	Fail

Supervisor Name …………………………………… Sign……………………………………

***Show to midwife teacher**

Midwifery Clinical Skill 2. Belly check. (Abdominal examination).	Yes	NA	No
Be kind to the woman, tell her your name. **Give Privacy**<br>Ensure bladder is empty. Explain to the woman what you will do			
Ask the woman if she feels well, is the baby moving and any complaints?			
Check CRF for correct EGA, any risk factors, previous obstetric history, morbidity sheet, weight gain, fundal height gain, fetal position and last BP.			
Check for any comments or doctor's orders written down on CRF.			
Student can take BP accurately and write down correctly in CRF.			
Ask the woman to lie down with pillow under her head, (left lateral tilt if needed). Examine her belly with sarong down.(if not private keep sarong over the belly after looking)			
**(Look)**. **Inspection:** Look for size, shape, skin condition, stretch marks, scars, linea nigra (dark line), and fetal movements.			
**FH**. Check fundal height correctly with a tape measure in centimeters from the top of the symphysis pubis to the top of the fundus.			
**(Feel) Palpation:** Use step-by-step movements to find out; lie, presentation, position, flexion, engagement.			
Estimate weight of fetus (kilo) and if water bag volume normal or not normal.			
**(Listen) Auscultation:**<br>Place fetal stethoscope correctly over the fetal heart and listen for one minute. **Student must be able to listen with Pinard stethoscope to pass belly check.**<br>Only use doppler so the mother can also hear FHB or if <24 EGA.			
Give results step by step, to supervisor: e.g. Fundal height in relation to EGA, fetal lie, presentation, position, flexion, engagement, fetal heart rate and regularity. Show with doll and pelvis, if available.			
Student can say if normal or not normal, and make a plan.<br>When asked, can state when to refer to senior midwife, medic or doctor.			
Write down everything correctly in CRF.			
Explain to the mother and encourage her to ask questions. Tell her where to wait for results and when to come back for next ANC visit			
First assessment after x3 with supervision from senior midwife or doctor. Can do step by step.<br>***Comments or feedback***	Date	Safe	***Not safe****
Supervisor Name …………………………………… Sign……………………………………			
Repeat assessment IF NEEDED after x 6 with supervision from senior midwife or doctor. Can do step by step.<br>***Comments or feedback.***	Date	Safe	***Not safe****
Supervisor Name …………………………………… Sign……………………………………			
Final assessment after at least 50 by yourself. Comments or feedback	Date	Pass	Fail
Supervisor Name …………………………………… Sign……………………………………			
Repeat final assessment IF NEEDED. Comments or feedback	Date	Pass	Fail
Supervisor Name …………………………………… Sign……………………………………			

***Show to midwife teacher**

Midwifery Clinical Skill 3. Vaginal examination for Bishop's score or membranes sweep.	Yes	NA	No
**BE KIND** to the woman, tell her your name. **Give Privacy**<br>Ensure bladder is empty. Explain to the woman what you will do. Ask for her consent.			
Can give the reason for VE and membrane sweep or induction of labour.			
Help the woman to get on the delivery bed.			
Do full belly check (abdominal examination) including FHB. Listen for one minute.			
Prepare equipment for VE, wash hands and wear non-sterile gloves.			
Check vulva vagina for any abnormalities and wash with Savlon if needed.			
**Bishop's score.** Insert two fingers gently into vagina, feel cervix and check for:<br>• Position,<br>• Consistency,(Hard or soft)<br>• Effacement, (shortening)<br>• Dilatation, (cm)<br>• Station of presenting part above or below ischial spines.			
Supervisor to double check VE.			
Calculate Bishop's score using the graph and give step-by-step handover to the supervisor.<br>Check FHB again			
**Make a plan.** *E.g. Wait for delivery or discuss with doctor.*			
**Sweeping of the membranes.**<br>***This is only done after discussion of Bishop's Score with a doctor or senior midwife.***			
Tell the woman she may feel a little pain. Wash hands, put on gloves.<br>Insert one finger gently into cervix as far as it will go and sweep the finger slowly in a full circle (360°) one way and then the other way. Repeat as needed.<br>Take off gloves and wash hands.			
Check FHB again. Listen for one minute.			
Tell the woman she may have a little mucous and blood loss or contractions.<br>**Tell her danger signs and when to come to hospital.**			
**Induction of labour (IOL) with misoprostol (Cytotec)**.<br>***This is only done after discussion of Bishop's Score with a doctor or senior midwife.***			
State the dose, contraindications and side effects of misoprostol for IOL.			
Before giving misoprostol, check vital signs, FHB and explain to the woman.			
Wash hands, put on gloves, Put misoprostol tablets high into the posterior vagina.<br>Cover the woman, take off gloves and wash your hands.			
Tell the woman to lie down for one hour and then start to walk around.			
Check FHB and any contractions after the insertion. Follow SMRU protocol			
Write down time of misoprostol insertion and vital signs on CRF.			
Help the woman to get off the bed. Clean all equipment and delivery bed.			
First assessment after x3 with supervision from senior midwife or doctor. Can do step by step.<br>***Comments or feedback***	Date	Safe	*Not safe**
Supervisor Name …………………………………… Sign……………………………………			
Repeat assessment IF NEEDED after x 6 with close supervision from senior midwife or doctor<br>***Comments and feedback***	Date	Safe	*Not safe**
Supervisor Name …………………………………… Sign……………………………………			
Final assessment after at least 10 by yourself. Comments or feedback	Date	Pass	Fail

Supervisor Name …………………………………… Sign……………………………………

***Show to midwife teacher**

Midwifery Clinical Skill 4. Vaginal examination in labour.	Yes	NA	No
**BE KIND** to the woman, tell her your name. **Give Privacy.**<br>Explain to the woman what you will do. Ask for her consent<br>Help the woman to get on the delivery bed (left lateral tilt if needed).			
Ensure empty bladder.			
Student can say the reason for VE, any risks of VE and any actions if they occur.			
Do full belly check (abdominal examination) including FHB. Listen for 1minute.<br>State the position, flexion and engagement of fetal head.			
Prepare equipment for VE, wash hands and wear non-sterile gloves .			
Check vulva, vagina for any abnormalities and wash with Savlon if needed.			
Insert two fingers gently into vagina, feel cervix and check for:<br>• Position,<br>• Consistency,(Hard or soft)<br>• Effacement, (shortening)<br>• Dilatation, (cm)<br>• Station of presenting part above or below ischial spines.			
Any moulding?			
Any caput?			
Feel for membranes (water-bag). Do they bulge with contractions?			
If membranes already ruptured, check for meconium.			
Supervisor double check VE.<br>Cover the woman, take off gloves and wash your hands.			
Give step-by-step handover to the supervisor.<br>Student can say if VE normal or not normal.			
Make a **plan of care**.<br>*E.g. wait for delivery, or augmentation of labour by AROM*			
When asked, can state when to refer to senior midwife, medic or doctor.			
Tell the woman what you felt on VE and give encouragement.			
Help her to get down off the table.			
Write down VE results on partogram or management sheet.			
Clean all equipment and delivery bed.			
First assessment after x3 with supervision from senior midwife or doctor. Can do step by step.<br>***Comments and feedback***	Date	Safe	*Not safe**
Supervisor Name …………………………………… Sign……………………………………			
Repeat assessment IF NEEDED after x 6 with close supervision from senior midwife or doctor<br>***Comments and feedback***	Date	Safe	*Not safe**
Supervisor Name …………………………………… Sign……………………………………			

***Final assessment after at least 30 by yourself.* Comments and feedback**	Date	Pass	Fail
Supervisor Name …………………………………… Sign……………………………………			

***Show to midwife teacher**

**Midwifery Clinical Skill 5. Speculum vaginal examination.**	**Yes**	**NA**	**No**
State the reason for speculum vaginal examination.<br>Ask for assistance from another staff member if needed.			
Empty bladder.			
BE KIND. Tell her what you will do. Ask for her consent, Give privacy. Help the woman to get on the delivery bed. (Left lateral tilt if needed).			
If the woman is >20 weeks EGA, Do belly check and FHB.			
Prepare equipment, strong light, wash hands and wear non sterile gloves, check speculum is working.			
Check vulva, vagina for any abnormalities and wash with Savlon if needed			
Put clean water or jelly on speculum			
Gently separate the labia with 2 fingers. Tell the woman you will put the speculum into her vagina and help her to relax pelvic floor muscles.			
Insert the speculum gently, (held sideways-handle lateral) in the direction of the woman's back.			
As it is inserted turn it 90 degrees (handle posterior)			
Slowly open the blades to show the cervix. (if any pain, stop and gently try again)			
Lock the blades and look for cervix and posterior fornix.<br>Check for<br>• Cervix dilatation, colour, any abnormalities.<br>• Discharge in the vagina/candida patches/bad smell<br>• Water bag leaking or mucous from cervix.<br>• Blood, blood clots from cervix<br>• Any tissue or placenta in cervical os.<br>• Any other objects, e.g. bamboo, leech.			
Remove any products/clots with sponge forceps, gauze or fingers.<br>Examine products removed to identify fetal parts or placenta & membranes.			
Take swabs if needed. **Supervisor to double check as needed.**			
Unlock speculum and gently remove from vagina			
Cover the woman, take off gloves and wash your hands.			
Give step-by-step handover to the supervisor. Make a plan for care.			
Write down VE on CRF, partogram or management sheet.			
When asked, can state if any abnormal findings and when to refer to doctor.			
Tell the woman what you felt on VE. Help her to get down off the table.			
Clean all equipment and delivery bed.			
First assessment after x3 with supervision from senior midwife or doctor. Can do step by step.<br>***Comments and feedback***	Date	Safe	*Not safe**
Supervisor Name …………………………………… Sign……………………………………			
Repeat assessment IF NEEDED after x 6 with close supervision from senior midwife or doctor<br>***Comments and feedback***	Date	Safe	*Not safe**
Supervisor Name …………………………………… Sign……………………………………			
Final assessment after at least 10 by yourself. Comments and feedback	Date	Pass	Fail
Supervisor Name …………………………………… Sign……………………………………			

***Show to midwife teacher**

Midwifery Clinical Skill 6. Normal delivery (NVD).	Yes	NA	No
**Before doing any delivery, can do role play with no mistakes**			
Before delivery, call for help and prepare birth equipment.			
Review any risk factors, e.g. for PPH, shoulder dystocia, neonatal resuscitation.			
Check if mother has empty bladder. Do belly check for fetal position, engagement.			
**BE KIND**. Tell the woman what to do. Encourage upright positions.			
When head is on view, put on apron, goggles .Wash hands, put on non-sterile gloves, open delivery box, prepare for delivery.			
Support perineum correctly during crowning, extension and birth of head.<br>Tell mother to stop pushing if needed to slow down delivery of the head.			
Make sure chin is over the perineum. Check for cord around the neck.			
Watch for restitution. Wipe any mucous or blood away from the baby's face.			
Watch for external rotation.			
Help with delivery of the shoulders. Put hands on each side of the fetal head and use gentle traction towards the sacrum to free the anterior shoulder. Use traction towards symphysis pubis to deliver the posterior shoulder.<br>WATCH perineum carefully and tell mother to stop pushing when needed.			
Tell midwife to give mother Oxytocin (Synto) 10 IU, IM with anterior shoulder.			
Hold the baby under the shoulders. Deliver the body by the curve of the birth canal.			
Watch the time of delivery. Give the baby to midwife to cut the cord.			
Put large bowl near the perineum. Put cord clamp close to perineum.<br>Put hand on the top of the uterus (fundus) to feel for contraction.			
Watch for signs of separation and descent of placenta.<br>*Uterus contracts and feels smaller and higher gush of blood at the vagina, cord comes out of vagina, mother wants to push.*			

Midwifery Clinical Skill 6. Normal delivery (NVD). (Continued)	**Yes**	**NA**	**No**
Check for contraction. Put one hand above symphysis pubis in the correct position. Hold the cord and use continuous traction towards the sacrum for 2-3 minutes or until you can see the placenta.			
Lift the cord towards the symphysis pubis to follow the curve of the birth canal to deliver placenta.			
Hold the placenta in both hands and twist the membranes to make them strong.			
Put placenta and membranes into the bowl. Watch time of placenta delivery.			
Check for contraction of uterus. *Gently rub up a contraction if needed.*			
Check the perineum and vagina for any tears–suture if needed.			
Help the woman to hold and feed her baby when she is ready (<1 hour).			
Check if placenta and membranes are complete, weigh placenta, measure blood loss (including from bucket) and take study samples if needed.			
Write down everything on partograph, outcomes sheet and log book after delivery.			
Help the woman to go to postnatal. Clean all equipment and delivery bed			
First assessment after x 3 with close supervision from senior midwife or doctor. ***Comments or feedback.***	Date	Safe	***Not safe****
Supervisor Name …………………………………… Sign……………………………………			
Repeat assessment IF NEEDED after x 6 with close supervision from senior midwife or doctor. ***Comments or feedback.***	Date	Safe	***Not safe****
Supervisor Name …………………………………… Sign……………………………………			
Final assessment after at least 20 by yourself. Comments or feedback	Date	Pass	Fail
Supervisor Name …………………………………… Sign……………………………………			
Repeat Final assessment IF NEEDED. Comments or feedback	Date	Pass	Fail
Supervisor Name …………………………………… Sign……………………………………			

***Show to midwife teacher**

Midwifery Clinical Skill 7. Reception of the newborn.	**Yes**	**NA**	**No**
List all equipment needed for reception and resuscitation of the newborn.			
Check all equipment is ready and working correctly			
Explain to the woman what you will do for her baby after delivery.			
Wash hands; wear apron, goggles and sterile gloves.<br>Watch for time of delivery.			
At delivery, clamp and cut the cord safely then wrap the baby in a clean cloth.			
If baby is breathing well and no resuscitation is required, dry baby and keep warm.			
If baby is not breathing and/or has thick meconium, clamp and cut the cord quickly, do not dry or stimulate the baby. Follow resuscitation of newborn drill.			
Throw away wet cloth and cover baby with a clean cloth. Keep baby warm and safe.			
**Check Apgar Score at 1 minute**.			
Using aseptic technique, clamp/tie cord safely 2-3 cm from skin margins.			
**Check Apgar Score at 5 minutes**			
Accurately set up the baby scales. Weigh the baby naked. Check with another midwife.			
Accurately measure length (L), arm circumference (AC) and head circumference (HC).			
Check the baby from head to toe, step by step, for any obvious abnormalities.			
**15 minutes after delivery do newborn vital signs. Use MACHO to assess each baby.**<br>Check skin colour and baby appearance.			
Take the temperature correctly per rectum.			
Accurately count the baby's heart rate (HR) and respiratory rate (RR) for 1 minute each.			
Can state indications for Vitamin K and give IM injection safely.			
Help the mother to breastfeed ≤1 hour after delivery. **Check if any study samples needed.** Keep the baby with the mother until both are ready to go to postnatal area.			
When asked, **can give each step for APGAR score and MACHO, normal ranges for vital signs and when to refer to senior midwife, medic or doctor**. **Can make a plan for care.**			
Write down all newborn care correctly in CRF, outcomes sheet and delivery log book.			
Clean all equipment check it is working and put back in correct place.			
First assessment after x 3 with close supervision from senior midwife.<br>***Comments and feedback.***	Date	Safe	*Not safe**
Supervisor Name …………………………………… Sign……………………………………			
Repeat assessment IF NEEDED after x 6 with close supervision from senior midwife.<br>***Comments and feedback.***	Date	Safe	*Not safe**
Supervisor Name …………………………………… Sign……………………………………			
Final Assessment after at least 20 by yourself. Comments and feedback	Date	Pass	Fail
Supervisor Name …………………………………… Sign……………………………………			
Repeat Final Assessment if needed. Comments and Feedback	Date	Pass	Fail
Supervisor Name …………………………………… Sign……………………………………			

***Show to midwife teacher**

**Midwifery Clinical Skill 8. Resuscitation of the newborn. (Role play).**	**Yes**	**NA**	**No**
List all equipment needed for reception and resuscitation of the newborn.			
Check all equipment is ready and working correctly.			
State why a baby would need resuscitation at birth. *E.g. maternal or fetal problems*			
When given a scenario, can show using role play, and give reason for each action			
• Prevention of heat loss (dry baby, cover, heat source)			
• Initial assessment of HR breathing colour			
• Correct suction technique if thick meconium and baby not breathing (mouth first).			
• How to assess HR.			
• Correct positioning of baby (sniffing position), for airway maintenance			
• Demonstrate how to do cardiac massage (CPR) using ratio of 3 compressions to 1 breath at rate of 100-120 bpm.			
• State correct dose of **Adrenalin,** 1:1,000, **0.3cc.** (SC or IV injection).			
**Baby breathing well**<br>• HR >100 bpm, Colour pink<br>o Dry with warm towel<br>o Assist to breastfeed.			
**Baby not breathing well, colour not pink.**<br>• HR >100 bpm<br>• Already breathing no need to suck meconium.<br>o Dry with warm towel<br>o Give O_2 at 5L per min via face mask			
**Baby not breathing at all.**<br>• HR< 100 bpm, Colour pale or all blue<br>o Suck meconium if needed. Stop suction after 1 minute or if HR<60<br>o Dry baby with warm towel<br>o CALL FOR HELP-**Emergency**			
o Give 5 inflation breaths<br>o **Reassess for HR, colour, breathing (**at 30 seconds)<br>o HR < 60 bpm start CPR 3 compressions: 1 breath. Insert IV line<br>o HR > 60 and not breathing, give breaths 60 per minute			
o **Reassess HR, colour, breathing**<br>o HR < 60 bpm, adrenaline 0.3 cc. Check dextrose.<br>o CPR for 5 mins			
o **Reassess HR, colour, breathing**<br>o HR < 60 bpm repeat adrenaline.<br>o **Reassess every 5 mins HR, colour, breathing**<br>o After 20 mins of no response-STOP.			
**Can give each step of MACHO and use it to assess every baby.**			
Give step-by-step handover to supervisor and make a plan for care.			
Say where to write down all actions (*in CRF, partogram and log book.)*			
Clean all equipment and put everything back in the correct place after resuscitation.			
First assessment Comments and feedback	Date	Pass	Fail
Supervisor Name …………………………………… Sign……………………………………			
Second assessment. Comments and feedback	Date	Pass	Fail
Supervisor Name …………………………………… Sign……………………………………			
Third assessment. Comments and feedback	Date	Pass	**Fail***

Supervisor Name …………………………………… Sign……………………………………			

*** If still fail after 3 exams show to midwife teacher**

**Midwifery Clinical Skill 9. Postnatal discharge (DC).**	**Yes**	**NA**	**No**
**The student can check vital signs of mother and baby with no mistakes.**			
**The student can check baby head to toe, step by step for any abnormalities and accurately assess the baby's colour using Kramer's score**			
Before discharge of mother and baby, educate her (and her family) about:			
Hygiene for mother; e.g. hand-washing, clean clothes, perineal care.			
Hygiene for baby, eye care, cord care with povidine, skin care , clean clothes			
Keep baby safe. How to protect baby from insects or animals near the house.			
Education about postnatal changes,<br>• Breast changes, milk comes in, nipples tender.<br>• Vaginal bleeding changes from red to brown, then white<br>• Bladder and bowel function, check for any continence problems,<br>• Perineal healing, any pain, signs of infection, pelvic floor exercises.<br>• Abdominal muscles diastasis > 4cms, postnatal exercises.			
Check if she has family to help at home, e.g. her mother, advise need to rest — 45 days			
Maternal nutrition advice, anemia prevention,			
**Family planning advice, does she want more babies? Folate before another pregnancy. Help her choose a FP method and explain correctly how to obtain/use contraception.**			
**Vaccination** schedule for baby and where to go.			
Tell her to give only breastmilk for the first 6 months and to give breastmilk for at least 1 year. Tell her how to keep breastfeeding if she goes to work.			
How to tell if baby has enough milk in the first weeks.			
Baby feeds 8-12 times a day.<br>6-8 clear urine per day.<br>2-4 bowel motions per day.<br>Sleeps for some time between feeds.<br>Is alert and happy when awake.<br>Baby gains weight (150-200g per week)			
If she is not breastfeeding, send to PMTCT counselor for education about using powder milk			
Danger signs Mother: Tell her to come to SMRU immediately if any;<br>• Fever or pain anywhere, bad smell, too much bleeding, dizziness.<br>• Breast problems, dysuria, bowel problems, vomiting or diarrhoea<br>• Depression or psychosis, worried about baby or herself. Husband is violent.			
Danger signs Baby: Tell her to come to SMRU with baby immediately if ;<br>• Fever, sleepy, floppy or blue, fitting or abnormal movements, crying too much.<br>• Skin rash, pustules or spots, sticky eyes, nail infections, cord has bad smell.<br>• Jaundice, not sucking strong, not passing urine, stools or vomiting, diarrhoea.			
Ask if she has any questions (include baby's father or other relatives and TBA)			
Write down all discharge information in CRF and Lema if she needs follow-up.<br>Tell the mother when to come for follow-up. Check if she has transport.			
Ensure bed and any equipment is cleaned after discharge.			
First assessment after x 3 with close supervision from senior midwife or doctor<br>***Comments or feedback.***	Date	Safe	***Not safe****
Supervisor Name …………………………………… Sign……………………………………			
Repeat assessment IF NEEDED after x 6 with close supervision from senior midwife or doctor.<br>***Comments or feedback.***	Date	Safe	***Not safe****
Supervisor Name …………………………………… Sign……………………………………			
Final Assessment after at least 20 by yourself. Comments or feedback	Date	Pass	Fail
Supervisor Name …………………………………… Sign……………………………………			

***show to midwife teacher**

**Midwifery Clinical Skill 10. Handover of cases to doctor, medic or senior midwife.**	**Yes**	**NA**	**No**
**If it is an emergency:** state clearly EMERGENCY at the start.			
Tell the doctor your name. Speak clearly and slowly in English.<br>Use these 4 steps to present information.<br>a) Case presentation (story) .Any risk factors<br>b) Examination and any test results<br>c) Diagnosis<br>d) Plan of care/ medications			
Say what has happened over the last hours/days.<br>**For a woman in pregnancy, labour or postnatal, the student can state:**<br>Listen carefully to any questions. Ask the supervisor to say it again or more slowly if you cannot understand<br>Tell the supervisor the reason you are telephoning. *E.g. the patient is now fully dilated but no progress for 2 hours.*			
If it is a new patient, give a **case presentation (tell the story)** from the start.			
Explain any signs and symptoms. E.g. test results, vital signs, vaginal bleeding, labour pain, abnormal findings.			
If it is not a new patient, give a brief **case presentation** (tell the story) and say what has happened since the last consultation.			
State the **diagnosis** or the problem.			
Make a **plan of care** including any medication.<br>Listen to any questions the doctor asks.			
Write down any instructions in the telephone book.<br>Write down any treatment or medicines on the CRF or IPD chart.<br>Explain any treatment to the woman and to other staff.			
**For a baby, the student can** Use the 4 steps to present information**:**<br>State the current age of baby in hours or days, type of delivery, Apgar scores and any resuscitation at birth.			
Give a brief history of any known maternal risk factors and what has happened since birth.			
State baby's vital signs, colour, any test results and the **reason for referral** to medic or doctor, weight, any weight loss since birth any feeding problems,			
When asked, can formulate a plan for care of this baby.			
Write any instructions or medication orders in the CRF straight away.			
Explain any treatment to the woman and to other staff.			
First assessment after x 3 with close supervision from senior midwife or doctor<br>***Comments and feedback.***	Date	Safe	***Not safe****
Supervisor Name …………………………………. Sign………………………………….			
Repeat assessment IF NEEDED after x 6 with close supervision from senior midwife or doctor. Comments and feedback.	Date	Safe	***Not safe****
Supervisor Name …………………………………. Sign………………………………….			
Final Assessment after at least 20 by yourself. Comments and feedback	Date	Pass	Fail
Supervisor Name …………………………………. Sign………………………………….			

***show to midwife teacher**

Reflection on practice instructions.

Reflection is thinking about something you did (e.g. belly check or normal delivery) and what was good and what was not good and then planning to make it better next time.

It is about what you experienced, and how you felt.

If you reflect (look back) when you made a mistake and when you made no mistakes and then make a plan for how to do better next time, you will learn very quickly.

Example of a reflection on practice.

- **Plan** (e.g. belly check) or something happened (e.g. PPH)

I was afraid because I had not done belly check before.

I asked the *senior midwife to help me. She was kind and showed me how to do it. Then I did not feel afraid*

- **Actions.** What did I do?

My supervisor helped me in ANC and I practiced belly check many times.

I studied hard from the SMRU Obstetric Manual

- **Evaluation**. Did I make any mistakes?

My supervisor helped me many times and now I can do belly check with no mistakes. Sometimes I cannot count the FHB because it is very fast.

- **Reflection.** How can I do this better?

I will keep practicing so I never forget how to do belly check and FHB

Now I feel confident to count the FHB.

Write down 3 examples of reflection on practice*.*

We will practice in class so don't worry if you do not know how to do it.

Reflection 1(thinking about what you do)

Plan____

Action____

Evaluation____

Relection____

Reflection 2 (thinking about what you do)

Plan____

Action____

Evaluation____

Relection____

Reflection 3 (thinking about what you do)

Plan____

Action____

Evaluation____

Relection____

Case study instructions.

Ask a woman if you can follow her pregnancy, delivery and PNC as a "Guardian Angel".

- EGA <30 April 2011.
- Can be a friend or sister or she may live in your village.
- Ask for consent and tell her she can stop any time.
- Write down your name and MIDWIFE STUDENT CASE STUDY on the CRF.
- If you have a mobile number, write it down on the CRF.
- Ask the staff to call you if the woman is admitted.
- Tell the woman to call you if she comes to clinic for any reason.

CONFIDENTIALITY.

- Keep your log book in a safe place.
- Do not let anyone at home read your log book.
- Do not tell anyone else about the woman (you can only talk to medical staff at the clinic).
- Do not write down the real name of the woman, only a nick-name.
- Write down only her ANC code.

PROFESSIONAL BEHAVIOUR

- Be kind and always give privacy.
- Do not give or accept money or valuable presents (small presents are OK, eg cookies).
- If you go to her house, treat the family with respect.

Pregnancy.

Every time she comes to ANC;

- Go to ANC and do belly check
- Check ANC card if any abnormality, make a plan
- Give her education, advice about everything.
- Write down in your log book everything that happened each week
- If she has any questions write down your answers in your log book.

Delivery

- Check with senior midwife and help to look after her
- If possible, stay with the woman during labour and delivery.
- Be kind and give support to woman and husband or family.
- Write down everything from the beginning
- If possible, deliver her baby, or cut the cord.
- After delivery do vital signs and help with the first breastfeed.

Postnatal (PNC).

- Write down daily check of the mother, any abnormalities, education, and advice.
- Write down daily checks of the baby, any abnormalities.
- If possible, do PNC discharge including education, vaccination, family planning, danger signs, any follow-up.

Home Visit.

- If possible, visit the family at home , one week after discharge
- Observe the mother and baby and the family together
- Write down what happened and any problems.
- Say thank you to the mother for helping you to be a midwife.

Case Study Assessment

You must complete the case study to pass the course.

If the woman is transferred to a Thai hospital, ask the supervisor or midwife teacher for help.

The midwife teacher will look at your case study at any time during the training, so keep it up to date.

Write down everything here, it does not matter if you make a mistake.

Case Study ANC code **Name (ONLY nickname)**

Pregnancy.

Full social, obstetric and medical history, first visit, EGA, risk factors, any problems and what happened.

Write down all her ANC visits and all care at each one. If **absent, why? If not normal-explain.**

Date,	EGA	Wt (Kg)	(FH)	Fetal position	FHB	BP	Temp	HCT	MS	Any Abnormalities, advice, education.

Date,	EGA	Wt (Kg)	(FH)	Fetal position	FHB	BP	Temp	HCT	MS	Any Abnormalities, Advice, Education.

Delivery.

Post Natal Care

Home Visit

Add more pages if you need here
